# ZNF184 negatively regulates HR repair and predicts poor prognosis in acute lymphoblastic leukemia

**DOI:** 10.1093/nar/gkag486

**Published:** 2026-05-20

**Authors:** Won Chan Hwang, Hee Young Ju, Kibeom Park, Eun Jung Kwon, Eun Seop Seo, Yuheon Chung, Byung-Gyu Kim, Kyungjae Myung, Dong Min Lim, Yun Hak Kim, Keon Hee Yoo, Hongtae Kim

**Affiliations:** Department of Biological Sciences, Ulsan National Institute of Science and Technology, Ulsan 44919, Republic of Korea; Department of Pediatrics, Samsung Medical Center, Sungkyunkwan University School of Medicine, Seoul 06351, Republic of Korea; Department of Biological Sciences, Ulsan National Institute of Science and Technology, Ulsan 44919, Republic of Korea; Medical Research Institute, Pusan National University, Yangsan, Gyeongsangnam-do 50612, Republic of Korea; Department of Pediatrics, Samsung Medical Center, Sungkyunkwan University School of Medicine, Seoul 06351, Republic of Korea; Department of Health Science and Technology, SAIHST, Sungkyunkwan University, Seoul 06351, Republic of Korea; Samsung Genome Institute, Samsung Medical Center, Seoul 06351, Republic of Korea; Center for Genomic Integrity, Institute for Basic Science, Ulsan 44919, Republic of Korea; Center for Genomic Integrity, Institute for Basic Science, Ulsan 44919, Republic of Korea; Center for Genomic Integrity, Institute for Basic Science, Ulsan 44919, Republic of Korea; Department of Biomedical Engineering, Ulsan National Institute of Science and Technology, Ulsan 44919, Republic of Korea; Interdisciplinary Program of Genomic Data Science, Pusan National University, Yangsan, Gyeongsangnam-do 50612, Republic of Korea; Department of Biomedical Informatics, School of Medicine, Pusan National University, Yangsan, Gyeongsangnam-do 50612, Republic of Korea; Department of Anatomy, School of Medicine, Pusan National University, Yangsan, Gyeongsangnam-do 50612, Republic of Korea; Research Institute for Convergence of Biomedical Science and Technology, Pusan National University Yangsan Hospital, Yangsan, Gyeongsangnam-do, 50612, Republic of Korea; Department of Pediatrics, Samsung Medical Center, Sungkyunkwan University School of Medicine, Seoul 06351, Republic of Korea; Department of Health Science and Technology, SAIHST, Sungkyunkwan University, Seoul 06351, Republic of Korea; Cell & Gene Therapy Institute, Samsung Medical Center, Seoul 06351, Republic of Korea; Department of Biological Sciences, Ulsan National Institute of Science and Technology, Ulsan 44919, Republic of Korea

## Abstract

Zinc finger proteins (ZNFs) are increasingly recognized as regulators of oncogenic transcriptional networks and DNA damage responses. Through integrative analysis of bulk and single-cell RNA sequencing data, we identified a conserved set of seven ZNF genes, including ZNF184, that are upregulated in acute lymphoblastic leukemia (ALL) and exhibit dynamic expression patterns linked to disease progression. Among these, ZNF184 uniquely localized to DNA double-strand breaks (DSBs) in a zinc finger domain-dependent manner. Functional analyses revealed that ZNF184 suppresses homologous recombination (HR)-mediated DNA repair by impeding BRCA1 recruitment, leading to accumulation of DNA damage. ZNF184 expression was elevated in primary ALL samples and associated with increased γH2AX levels and inferior overall survival in ALL patients. Loss of ZNF184 restored HR efficiency, reduced DNA damage burden, and enhanced genome stability, while re-expression re-sensitized cells to DNA-damaging agents. Mechanistically, ZNF184 directly interacted with TRIM28 and facilitated its recruitment to DSBs, modulating TRIM28 phosphorylation and chromatin remodeling through the HP1/SUV39H1 complex. ZNF184 expression conferred heightened sensitivity to PARP inhibition and synergized with genotoxic chemotherapy in both cell lines and patient-derived ALL cells. These findings identify ZNF184 as a key modulator of DSB repair and a predictive biomarker for therapeutic strategies targeting HR-deficient ALL.

## Graphical Abstract

**Graphical Abstract:**
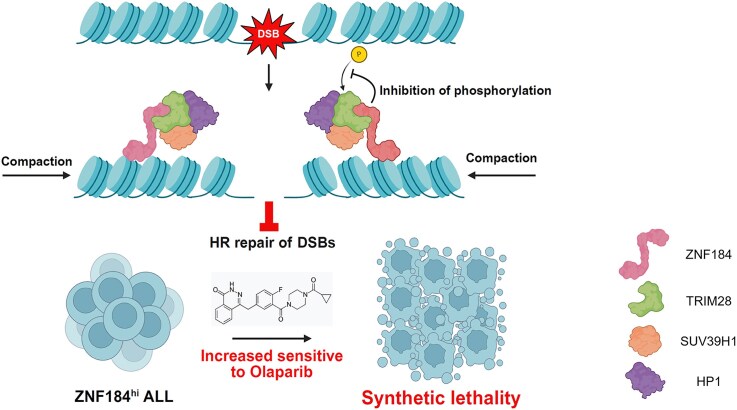

## Introduction

Acute lymphoblastic leukemia (ALL) is the most common malignant disease in children, arising from heterogeneous factors including germline predisposition, infection, and environmental toxins [1, 2]. There are several well-known recurrent genetic abnormalities associated with childhood ALL, which can be pathogenic and deciding the outcome of ALL [1]. In recent years, the development of genetic testing has led to the identification of previously unknown genetic abnormalities associated with ALL, such as Philadelphia-like ALL, *TCF3::HLF*, and *ZNF384* rearrangements [3–5]. Among these novels ALL subtypes that are distinguished from the former known recurrent genetic abnormalities, the role of the zinc finger domain has gained attention with the discovery of *ZNF384* fusion [6–8].

The DNA damage response (DDR) is a conserved network crucial for maintaining genomic stability [9]. Among various types of DNA damage, double-strand breaks (DSBs) are especially harmful, repaired mainly by nonhomologous end joining (NHEJ) or high-fidelity homologous recombination (HR) [10–13]. Defects in these pathways can lead to mutations or cell death. Synthetic lethality refers to a condition in which the simultaneous loss of two genes leads to cell death, whereas the loss of either gene alone is tolerated [11, 14–16]. Poly (ADP-ribose) polymerase inhibitors (PARP inhibitors, PARPi) exploit HR deficiencies in BRCA1/2-mutant cancers, selectively targeting tumor cells. Ongoing research continues to uncover new synthetic lethal interactions for targeted treatments [9, 14–17].

Zinc finger proteins (ZNFs) are a diverse group involved in key cellular processes such as transcriptional regulation, chromatin remodeling, hematopoiesis, and DNA repair [18, 19]. ZNF184, a predicted member of the Kruppel C2H2-type ZNF family, contains N-terminal Krüppel-associated box (KRAB) domains and C-terminal zinc finger motifs. Genome-wide studies have linked ZNF184 to certain genetic diseases [20, 21]. KRAB domains typically recruit TRIM28 [Tripartite motif-containing 28, also known as KRAB-associated protein-1 (KAP-1)], a transcriptional corepressor involved in heterochromatin formation, DDR, p53 inhibition, EMT, stem cell maintenance, and genomic stability [22, 23]. TRIM28 functions are modulated by posttranslational modifications like phosphorylation and SUMOylation [22]. Although ZNF184 is presumed to regulate transcription via TRIM28 binding, its specific target genes and role in the DDR remain unclear.

In this study, we identify ZNF184 as a negative regulator of the DDR pathway, with significant implications for DSB repair and hematologic malignancies. ZNF184 is recruited to DNA damage sites through its zinc finger domain, and its overexpression suppresses HR repair. Elevated ZNF184 expression is associated with the accumulation of DSBs, increased γH2AX levels, and reduced survival in ALL patients. Mechanistically, ZNF184 impairs BRCA1 recruitment to DSB sites, enhancing genomic instability and sensitizing cells to ionizing radiation. Moreover, ZNF184 overexpression sensitizes tumor cells to PARPi such as Olaparib, making it a potential therapeutic target. We also identify TRIM28 as a novel ZNF184 interactor, demonstrating their coordinated recruitment to DNA damage sites and TRIM28 phosphorylation modulation by ZNF184. Together, these findings establish ZNF184 as a critical mediator of DDR and a promising target for improving therapeutic strategies in ALL.

## Materials and methods

### Human specimen collection

Bone marrow (BM) and/or peripheral blood samples were obtained from patients with precursor B-cell ALL at diagnosis, relapse, and remission. Extra BM specimens were collected and processed in accordance with the institute’s protocol during BM examination. BM specimens were stored at the Samsung Medical Center Biobank after the following preparation process. This study was approved by the Institutional Review Board of Samsung Medical Center (approval No. 2023-03-041-011). Patient consent was obtained in accordance with the Declaration of Helsinki.

### Human sample preparation

BM samples were collected in RPMI-1640 medium supplemented with 50 IU/ml heparin. To remove red blood cells (RBCs), the collected BM samples were treated with RBC Lysis Buffer (BioLegend). For *in vivo* experiments, BM samples were processed immediately after collection and used in a fresh state without cryopreservation, in order to preserve cellular viability and physiological characteristics. For other applications, the processed samples were cryopreserved using CELLBANKER^®^ 1 (NIPPON ZENYAKU KOGYO CO.) and stored in liquid nitrogen.

### Single cell RNA sequencing analysis

Single-cell RNA sequencing data (GSE130116) [24] was downloaded from the Gene Expression Omnibus (GEO, https://www.ncbi.nlm.nih.gov/geo/). For this study, only samples from B-ALL with *ETV6::RUNX1* fusion patients and healthy donors were included. The methodologies used in this study are consistent with those described in our previous publication [25–27]. All analyses were performed using the “Seurat” package (v4.3.0) [28] in R (v4.2.2), with default parameters applied unless otherwise specified. High-quality data were obtained through cell- and gene-level filtering to ensure suitability for analysis. The criteria for exclusion were as follows: (i) Cells with fewer than 1000 unique molecular identifiers (UMIs); (ii) Cells with fewer than 500 or >4000 detected genes; (iii) Cells with reduced RNA complexity, reflected by a gene-to-UMI ratio (log_10_-transformed) of <0.8; (iv) Cells with mitochondrial gene expression exceeding 10%; and (v) Genes expressed in fewer than 10 cells.

Raw read counts were log-normalized, and highly variable features were identified using the “vst” method, selecting the top 2000 most variable features. Batch correction and data integration across samples were performed with the canonical correlation analysis algorithm. The integrated data were scaled, and dimensionality was reduced to 50 principal components. Clustering was conducted based on a shared nearest-neighbor graph (resolution = 1.0 for healthy/diagnosis samples; 2.5 for healthy, diagnosis, remission, and relapse samples). Clusters were represented in two dimensions using Uniform Manifold Approximation and Projection (UMAP). B cells were identified using established marker genes, including CD79A, CD19, MME, and CD24 [24]. To focus on the ZNF gene family, differentially expressed genes (DEGs) were identified from a subset of ZNF genes extracted from the whole transcriptome in B cells. The analysis was performed using the FindMarkers function in the “Seurat” package, applying thresholds of |log_2_FC| > 0.3 and adjusted *P*-value <.05.

### Bulk RNA sequencing analysis

RNA-Seq transcriptome and clinical data from the GSE116229 dataset [29] were downloaded and analyzed using R (v4.2.2). Differential expression analysis between conditions was conducted with the “limma” package (v3.48.3) [30], and was restricted to ZNF genes. Genes with an adjusted *P*-value <.05 were considered significantly differentially expressed.

### Bone marrow sampling and whole transcriptome sequencing

From July 2023 onward, we prospectively collected BM specimens from pediatric patients with hematologic malignancies and from individuals undergoing BM evaluation for other clinical indications, under approval from the Samsung Medical Center Institutional Review Board (IRB No. 2023-03-037). Parents or legal guardians provided written informed consent for tumor sequencing and prospective collection of clinical information from medical records. Total RNA was isolated using either the PAXgene Blood RNA Kit (PreAnalytiX) or the High Pure RNA Isolation Kit (Roche). RNA quantity was measured with a Qubit fluorometer, and integrity and purity were assessed on an Agilent Bioanalyzer. Stranded whole-transcriptome libraries were prepared with the Illumina Stranded Total RNA Prep with Ribo-Zero Plus kit and sequenced on an Illumina NovaSeq 6000 platform (2× 100-bp paired-end). We targeted >100 million reads per sample (mean ~130 million). Reads were aligned to the hg19 reference genome using STAR1 (v2.5.2b) [31]. Gene-level transcript abundance was estimated with RSEM2 (v1.3.0) [32] to generate TPM values.

### Survival analysis

Gene expression and clinical data from the TARGET-ALL-P2 cohort [33] were retrieved using the “TCGAbiolinks” (v2.26.0) [34] R package, followed by survival analysis using the “survival” (v3.4.0) [35] and “survminer” (v0.4.9) [36] R packages. Patients were stratified into high- and low-expression groups based on the mean ZNF184 expression level. Kaplan–Meier survival curves were generated, and group differences were assessed using the log-rank test.

### Whole genome sequencing data preprocessing

We initially assessed the quality of paired-end read files produced by the Element AVITI sequencing platform using FastQC (v0.11.9) (https://www.bioinformatics.babraham.ac.uk/projects/fastqc). Cutadapt (v3.6) [37] was utilized to trim adapter contamination and remove low-quality sequences. Trimmed reads with a minimum Phred quality score of 30 at the 3′ end, and a read length of at least 70 base pairs were retained, as BWA-MEM [38] performs optimally with reads of this length range (70–100 bp, FastQC). The alignment of trimmed reads to the human reference genome GRCh38 was conducted using BWA-MEM (v0.7.15), with the -M flag for Picard compatibility. Duplicate reads in the mapped dataset were eliminated using MarkDuplicatesSpark in GATK4 (v4.2.4.1). CountBasesSpark in GATK4 was employed to calculate the total number of bases, ensuring sufficient read-depth coverage (at least 85 gigabases) for detecting genetic variations. Excluding chromosome M, we focused on autosomal chromosomes 1–22 and sex chromosomes X and Y during this step. Subsequently, base quality scores were generated using BaseRecalibrator in GATK4 to assess the accuracy of each base call. ApplyBQSR in GATK4 was then used to apply base quality score recalibration, aiming to identify and rectify systematic errors introduced by the sequencing platform.

### Analysis of somatic variation

We conducted whole-genome sequence (WGS) analysis following GATK4 best practices for short somatic variant discovery [39]. In brief, we utilized Mutect2 in GATK4 to detect somatic variants, employing a “panel of normals” constructed from 1000 Genomes participants and leveraging the gnomAD database as a “germline-resource.” Our analysis incorporated all somatic variant calls that passed the standard Mutect2 filters. Specifically, we disregarded the “germline_risk” filter during Mutect2 analysis, including any calls where it was the sole filter applied. Subsequently, the identified somatic variant calls were annotated using FUNCOTATOR. Following variant calling and annotation, the mutation annotation file was imported into R using the “maftools” package (v3.14) [40]. All somatic variation analysis was performed using R (v4.1.3).

### Cell culture

Leukemia cell lines (REH, MOLT4, Jurkat, CCRF-CEM, and SUP-T1) and nonleukemia cell lines (HeLa and HEK293T) were used (American Type Culture Collection, ATCC). The REH, MOLT4, Jurkat, CCRF-CEM, and SUP-T1 cell lines were maintained in RPMI1640 (Welgene, LM011-01) supplemented with 10% fetal bovine serum (FBS, Gibco, A5670701) and 1% penicillin/streptomycin (Welgene, LS202-02) in 5% CO_2_ in a 37°C incubator. The HeLa and HEK293T cell lines were maintained in Dulbecco modified Eagle’s medium (Welgene, LM001-01) supplemented with 10% FBS and 1% antibiotic/antimycotic (Gibco, 15240062) in 5% CO_2_ in a 37°C incubator.

### Plasmids, sgRNA, siRNA

*ZNF184* D1, D2, D3, D4, and D5 deletion mutant expression plasmids were created using GFP- or Myc-tagged mammalian expression vectors. The *ZNF184* deletion mutants were generated from GFP-tagged *ZNF184* using Phusion Site-Directed Mutagenesis Kits (Thermo Fisher Scientific, F541). *ZNF184* genes were purchased from the Korea Human Gene Bank. GFP-, Flag-tagged *TRIM28*, ΔN-term, N-term, C-term, and M-term domain fragment deletion mutant expression plasmids were gifted from Prof. Kyungjae Myung (UNIST). *TRIM28* D1, D2, D3, and D4 deletion mutant expression plasmids were created using GFP- or Flag-tagged mammalian expression vectors. The *TRIM28* deletion mutants were generated from GFP-, Flag- or HA-tagged *TRIM28* using Phusion Site-Directed Mutagenesis Kits. mCherry-TRIM28 expression plasmid was created using mCherry-tagged vector. Guide RNA plasmids for human ZNF184 gene were generated by cloning guide sequences into pX330 (Addgene, #42230). Target sequences for gene editing were selected using SYNTHEGO (https://design.synthego.com). The primers for plasmids construction, siRNA, and sgRNA sequences used in this project are listed in the Supplementary Table S4.

### Antibodies

The following antibodies were used in the present study: anti-Flag-HRP (Sigma–Aldrich, A8592), anti-Myc-HRP antibody (Sigma–Aldrich, SAB4200742), anti-HA-HRP antibody (Roche, 12013819001), anti-Myc antibody (Sigma–Aldrich, SAB4501941, M5546), anti-GFP (Clontech, 632380), anti-BRCA1 antibody (Santacruz, SC-6954), anti-BRCA2 antibody (Sigma–Aldrich, OP95), RAD51 antibody (Abcam, ab63801), anti-β-actin antibody (Sigma, A5441), Anti-γH2AX antibodies (Cell signal, #2577), anti-ZNF184 antibody (Proteintech, 26100-1-AP), anti-TRIM28 antibody (BETHYL, A300-274A), anti-phosphoTRIM28 S824 (BETHYL, A300-767A), anti-phosphoTRIM28 S473 (Biolegend, 654102), anti-Rabbit IgG (H + L)-Alexa Fluor 594 (Thermo Fisher Scientific, A-11012), anti-Mouse IgG (H + L)-Alexa Fluor 594 (Thermo Fisher Scientific, A-11032), anti-Rabbit IgG (H + L)-Alexa Fluor 488 (Thermo Fisher Scientific, A-11008), anti-Mouse IgG (H + L)-Alexa Fluor 488 (Thermo Fisher Scientific, A-11001), and horseradish peroxidase-conjugated secondary antibodies specific to rabbit (Sigma–Aldrich, A0545) or mouse (Sigma–Aldrich, A9917) IgG.

### Generation of ZNF184 knockout cells

To generate the *ZNF184* knockout (KO) cell lines, REH cells were cotransfected with CRISPR/Cas9 and double guide RNA targeting *ZNF184* plasmids using the Neon Transfection system (Thermo Fisher Scientific). After 48 h, high GFP-expressing cells were sorted into 96-well plates using a FACSAria Fusion cell sorter (BD Biosciences). *ZNF184* KO cell lines were confirmed by targeted sequencing and western blotting for *ZNF184*. Single-cell-derived KO clones were isolated by limiting dilution and validated by Sanger sequencing and immunoblotting to avoid clonal heterogeneity.

### Generation of ZNF184 overexpressing cells in by lentiviral system

To generation of wild type (WT) or mutants ZNF184 overexpressing cell lines in REH-*ZNF184* KO cells, *ZNF184* WT and mutants were subcloned into lentiviral vector (pLenti CMV GFP 2A Puro vector, ABM, LV073) and cotransfected with pMD2.G, psPAX2 (Addgene, #12259, #12260) using Lipofectamine 3000 (Thermo Fisher Scientific, L3000015) into HEK293T cells. On the following day, transfected cells were refreshed with new medium, and lentivirus-containing medium was collected at 48 h and 72 h post-transfection. For viral transduction, REH-*ZNF184* KO cells were seeded into 6-well dish and infected for 24 h with virus-containing medium in the presence of polybrene (8 μg/ml, Sigma–Aldrich, H9268). To eliminate uninfected cells 24 h after infection, 0.1 µg/ml puromycin (InvivoGen, ant-pr-1) was added to the culture medium, and the cells were cultured for one week before being used in all subsequent experiments. The levels of overexpressed ZNF184 protein were confirmed by western blotting with anti-ZNF184 antibody.

### Immunofluorescence

For immunofluorescence colocalization with BRCA1, BRCA2, RAD51, Myc-tagging, γH2AX, and ZNF184 staining were performed as described previously with minor modifications [41]. Cells were fixed with 4% paraformaldehyde for 20 min at RT. After microirradiation cells were washed immediately PBS and fixed with 4% paraformaldehyde for 20 min at RT. All fixed cells were rinsed PBS two time, permeabilized with 0.5% Triton X-100 in PBS for 5 min at RT. Cells were then washed twice in PBS and incubated with blocking buffer (4% BSA in PBS) for 1 h at RT followed by incubation with primary antibody for overnight at 4°C. Slides and cells were washed three times with wash buffer (PBS containing 0.1% TritonX-100) and subsequently incubated with secondary antibodies conjugated with fluorophores (Alexa Fluor 594 or Alexa Fluor 488) and 4,6-diamidino-2-phenylindole (DAPI, 1 μg/ml, Thermo Fisher Scientific, D1306) for 1 h at RT. After three washes with wash buffer, slides were mounted using VectaMount AQ Aqueous Mounting Medium (Vector Laboratories, H-5501). Images were captured and analyzed using a LSM-880 confocal microscope. Nuclear fluorescence intensities were quantified in each sample using ZEN Blue software (Carl Zeiss).

### Transfection

For REH transfection, all transfected plasmids were added to 100 μl cell suspensions and electroporated using a Neon Tranfection System (Thermo Fisher Scientific, NEON1S) at 1350 V for 10 ms over three pulse. siRNAs were transfected into cells using Lipofectamine RNAiMAX reagent (Thermo Fisher Scientific, 13778150). Transient transfection in HeLa and HEK293T cells were performed by using poly(ethylenimine) (PEI, Polysciences, 24765) or Lipofectamine 3000. All experiments were performed at 48 h after transfection.

### Laser microirradiation and cell imaging

For laser microirradiation, cells were grown on 35 mm glass bottom dishes (SPL). HeLa cells were transfected with plasmids or siRNA for 24 h. Transfected cells were treated with 10 μM BrdU (Sigma–Aldrich) prior to laser microirradiation for 20 h. Laser-scanning confocal microscopy was performed using a Zeiss LSM880 microscope with a 40× W (N.A. 1.2) C-apo objective. DNA damage was induced in live cells (maintained at 37°C in a humidified environment at 5% CO_2_) using a 355-nm UVA optically pumped semiconductor laser (Coherent, Genesis, 15 µm/s, 100% power, 20 iterations). For GFP- and mCherry-tagged proteins, time-lapse images were acquired at 10 s time intervals after laser microirradiation. Acquisition and analysis were performed using ZEN software (Black edition, Zeiss).

### Cell proliferation assays

*ZNF184* KO cells were plated in 12-well plates. The number of living and dead cells was evaluated by Trypan Blue exclusion every 24 h for 72 h as previously described [42]. Cells were then counted every 24 h using a hemocytometer. Each experiment was repeated three times.

### Flow cytometry cell cycle assay

For EdU cell cycle analysis, Click-iT EdU Imaging Kit (Thermo Fisher Scientific, C10339) and BD Cytofix/Cytoperm™ Fixation/Permeabilization Kit (BD Biosciences, #554714) were used. *ZNF184* WT and KO cells were treated with EdU (5-ethynyl-2′-deoxyuridine) resulting in a final concentration of 10 μM and incubated for 1 h. Cells were harvested and washed with PBS twice. Cells were incubated at RT for 30 min with BD Cytofix/Cytoperm buffer, then washed twice with BD wash/perm solution diluted 10× in distilled water. Cells were permeabilized on ice for 20 min with BD Cytoperm™ Permeabilization Buffer Plus (BD Biosciences, # 561651) and washed with 1× BD wash/perm solution. Permeabilized cells were fixed again with BD Cytofix/Cytoperm buffer (BD Biosciences) for 10 min at RT and washed with PBS twice. The click-it reaction was performed according to the manufacturer’s instructions. DNA was stained with 1 µg/ml DAPI in DPBS for 1h at RT. Cells were analyzed using a BD FACSverse and data were analyzed in FlowJo v.10.8.1 (BD Biosciences).

### Liquid chromatography-tandem mass spectrometry based ZNF184 interactome analysis

To identify ZNF184 interactome proteins, HEK293T and REH cells were transfected with S-FLAG-streptavidin binding peptide (SFB)-tagged ZNF184 or control SFB vector plasmid. SFB-ZNF184 and associated proteins were immunoprecipitated from cell lysates using FLAG-M2 affinity gel (Sigma–Aldrich), and the immunoprecipitated proteins were separated by sodium dodecyl sulfate–polyacrylamide gel electrophoresis (SDS–PAGE). After staining with colloidal Coomassie blue, bands were sliced from the protein gel and in-gel tryptic digestion was performed as described by Shevchenko *et al*. [43]. Tryptic digests were analyzed using a Thermo Scientific Eazy-nano LC 1200 UHPLC system coupled with an Orbitrap Fusion Lumos mass spectrometer. Peptides were separated on a reversed-phase trap and analytical column and eluted with a gradient of 3%–80% acetonitrile over 80 min. MS data were acquired in data-dependent mode with a resolution of 120 000 for MS1 scans, selecting precursors with charges 2–7 and intensity >5e3 for fragmentation. Dynamic exclusion was set to 15 s with 10 ppm mass tolerance. MS/MS data were searched against the UniProt human database using the SEQUEST algorithm via Proteome Discoverer Sorcerer 2.1. Carbamidomethylation of cysteine was a fixed modification, while oxidation (M), N-terminal acetylation, and phosphorylation (S/T/Y) were variable. Results were validated with Scaffold (v4.10.0), accepting peptides with >97% probability and <1% FDR and proteins with >95% probability, <1% FDR, and at least two peptides, using ProteinProphet algorithm [44]. The mass spectrometry proteomics data have been deposited to the ProteomeXchange Consortium via the PRIDE [45] partner repository with the dataset identifier PXD (PXD066624).

### Immunoprecipitation and immunoblotting

For immunoprecipitation, cells were washed with ice-cold PBS and then lysed in NETN buffer (0.5% Nonidet *P*-40, 20 mM Tris, pH 8.0, 50 mM NaCl, 1 mM ETDA, 50 mM NaF, 100 µM Na3VO4, 1 mM dithiothreitol, and 50 µg/ml phenylmethylsulfonyl fluoride) with benzonase (Enzynomics, M018H) at 4°C for 120 min. Crude lysates were cleared by centrifugation at 14 000 rpm at 4°C for 15 min and supernatants were incubated with protein A-agarose-conjugated primary antibodies, FLAG-M2 affinity gel (Sigma–Aldrich, #A2220), or c-Myc agarose affinity gel (Sigma–Aldrich, #7470). Immunocomplexes were washed three times with NETN buffer and then subjected to SDS–PAGE. Western blotting was performed using the antibodies indicated in figure legends. Proteins were visualized using secondary horseradish peroxidase-conjugated antibodies and enhanced chemiluminescence reagent (Thermo Fisher Scientific). Signals were detected using an automated imaging system (ChemiDoc™; Bio-Rad Laboratories).

### Homologous recombination and nonhomologous end joining assay

The HR and NHEJ assay was performed as described previously with some adaptations [42]. U2OS DR-GFP or EJ5-GFP cells were transfected with the ZNF184 siRNAs in 12-well plates, and after 24 h, they were transfected with empty vector and I-SceI expression vector. Seventy-two hours after I-SceI transfection, cells were trypsinized, and the percentages of GFP positive cells were determined by flow cytometry.

### Statistical analysis

All results are based on two or three independent experiments and are presented as the mean ± standard deviation (SD). Comparisons between groups were performed using one-way or two-way ANOVA, *t*-test, and Wilcoxon rank-sum test. *P*-values <.05 were considered statistically significant. All statistical analyses were performed using Prism 10.5.0 (GraphPad). R software (v.4.2.2) was used to perform statistical analyses.

## Results

### Identification of ZNF genes implicated in the pathogenesis of acute lymphoblastic leukemia

**Figure 1.**
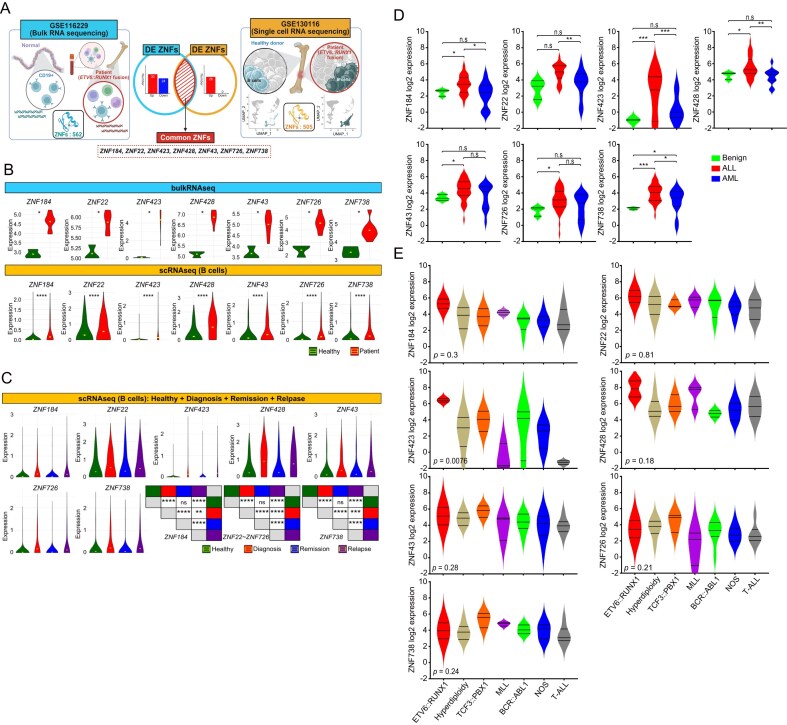
Identification of ZNF genes involved in ALL. (**A**) Overview of the process used to identify shared ZNF genes. The UMAP plots from single-cell RNA sequencing are color-coded to indicate B cells. Created in BioRender. Kwon, E. (2025) https://BioRender.com/f1ft8qw. (**B**) Violin plots showing the expression levels of the seven ZNF genes from bulk RNA sequencing (top panel) and in B cells from single-cell RNA sequencing (bottom panel). The yellow line within each plot represents the mean expression value. The *P*-value was calculated by Wilcoxon rank-sum test (*P < .05, ^****^P < .0001). (**C**) Violin plots and statistical comparisons of ZNF gene expression levels across healthy donors and ETV6-RUNX1-positive patients at diagnosis, remission, and relapse, based on single-cell RNA sequencing data. The yellow line within each violin plot represents the mean expression value. The accompanying heatmap summarizes statistical comparisons between groups for each ZNF gene. The *P*-value was calculated by Wilcoxon rank-sum test (**P < .01, ***P < .001, ^****^P < .0001, n.s., nonsignificant). (**D**) Expression levels of seven ZNF genes were represented as violin plots comparing Benign (*n* = 3) samples with ALL (*n* = 36) and AML (*n* = 12). The *P*-value was calculated by the unpaired t-test (*P < .05, **P < .01, ***P < .001, n.s., nonsignificant). (**E**) Violin plots were generated to show the expression levels of the seven ZNF genes across different ALL subtypes (ETV6::RUNX1; *n *= 2, Hyperdiploidy; *n *= 6, TCF3::PBX1; *n *= 3, MLL; *n *= 3, BCR::ABL1; *n *= 7, NOS; *n *= 9, T-ALL; *n *= 6). The *P*-value was calculated by the unpaired *t*-test.

ZNF proteins, a large family of transcription factors characterized by their zinc-coordinating DNA-binding motifs, are emerging as important regulators of oncogenic transcriptional networks. Recent studies have implicated various ZNF family members in hematologic malignancies, including ALL, where they may contribute to leukemogenesis and disease progression [46, 47]. However, the systematic identification of ZNF genes involved in ALL remains incomplete. To identify ZNF family genes potentially involved in ALL onset, we analyzed transcriptomic datasets encompassing both bulk and single-cell RNA sequencing. From bulk RNA sequencing data, 54 differentially expressed ZNF genes (DEGs) were identified out of 562 ZNF family members, with 30 upregulated and 24 downregulated genes in patient samples compared to healthy controls (Supplementary Table S1). Complementary analysis of single-cell RNA-seq data identified 26 upregulated ZNF DEGs among 505 genes specifically in *ETV6::RUNX1* fusion-positive ALL cases (Supplementary Table S2). Importantly, seven ZNF genes (ZNF184, ZNF22, ZNF423, ZNF428, ZNF43, ZNF726, and ZNF738) were commonly upregulated across both datasets (Fig. 1A and Supplementary Table S3). These seven genes exhibited significantly elevated expression in diagnostic ALL samples compared to healthy controls (Fig. 1B), indicating their potential involvement in the initial stages of leukemogenesis. Furthermore, Single-cell RNAseq data revealed dynamic changes in their expression levels over the course of disease. Expression of these ZNFs was markedly reduced during clinical remission and re-elevated upon relapse (Fig. 1C), suggesting a close association with disease activity and potential utility as molecular markers for disease monitoring. Validation using the BloodSpot database (www.bloodspot.eu) confirmed upregulation of six ZNFs in multiple ALL subtypes (Supplementary Fig. S1A–F). To improve the disease specificity and reliability of the selected ZNF genes associated with ALL, we analyzed RNA-seq data obtained from clinical patient samples. The expression levels of seven ZNF genes were compared across benign, ALL, and acute myeloid leukemia (AML) groups. Relative to benign samples, six of the seven genes-all except ZNF22-showed statistically significant differences in expression. When compared with AML samples, ZNF184, ZNF423, ZNF428, and ZNF738 were significantly upregulated in ALL (Fig. 1D). Furthermore, within the clinical ALL cohort, we assessed the expression of these ZNF genes across different ALL subtypes. Among them, ZNF423 exhibited a significant variation among subtypes, whereas the remaining six ZNF genes showed no subtype-specific differences (Fig. 1E). Collectively, these findings indicate that a group of ZNF-domain-containing genes is associated with leukemogenesis and disease progression.

### ZNF184 Is specifically recruited to DNA double-strand breaks via its zinc finger domain

**Figure 2.**
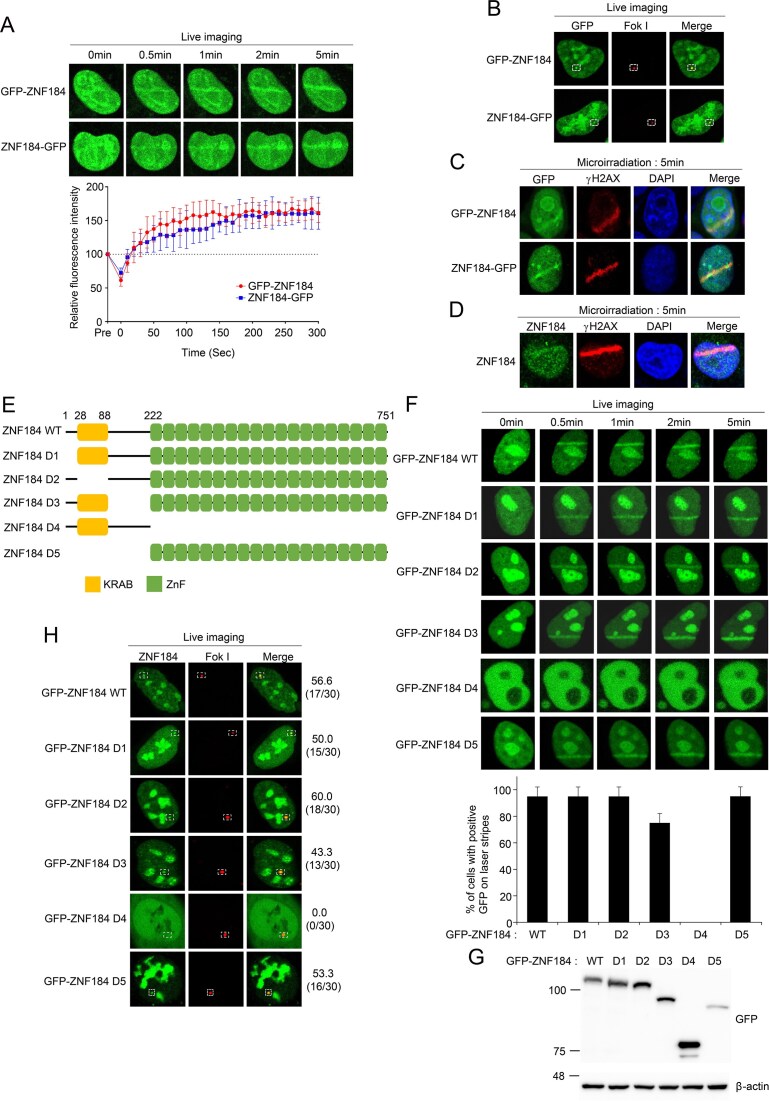
ZNF184 is recruited to the DSB sites through its ZnF domain. (**A**) HeLa cells expressing GFP-ZNF184 or ZNF184-GFP were subjected to laser microirradiation. The laser stripes were examined at the indicated time points. The intensity of each laser stripes in each time point was measured by averaging values from 10 cells and graphed in the lower panel. Data represents the mean ± SD of two independent experiments. (**B**) mCherry-LacI-FokI was cotransfected with indicated GFP-tagged expression vector into U2OS-DSB reporter cells. After 48 h of transfection, live cell imaging was performed with confocal microscopy. (**C**) HeLa cells expressing GFP-ZNF184 or ZNF184-GFP were subjected to laser microirradiation. After 5 min of laser microirradiation, the cells were fixed and stained with anti-GFP and -γH2AX antibodies. (**D**) HeLa cells were treated with laser microirradiation. After 5 min of laser microirradiation, the cells were fixed and stained with anti-ZNF184 and -γH2AX antibodies. (**E**) Diagram of WT ZNF184 and internal deletion mutants. Numbers indicate amino acids. (**F, G**) HeLa cells were transfected with GFP-ZNF184 WT or deletion mutant expression plasmids, and 24 h later, the cells were treated with laser microirradiation (top panel). The cells with positive GFP expression on the laser stripes are presented in the bar graph (bottom panel). The results represent the average of two independent experiments. The error bars indicate the SD for the cells transfected with each expression plasmid. (**H**) mCherry-LacI-FokI was cotransfected with indicated GFP-tagged expression vector into U2OS-DSB reporter cells. After 48 h of transfection, live cell imaging was performed with confocal microscopy. GFP-positive cells over total counts were denoted.

ZNF184, ZNF22, ZNF43, ZNF423, ZNF428, ZNF726, and ZNF738 are members of the Krüppel-type ZNFs family, a large class of transcriptional regulators involved in diverse cellular processes. Increasing evidence suggests that several ZNFs also participate in the DDR, although their specific functions in this context remain largely undefined [48, 49]. To investigate whether these ZNF proteins are recruited to DSBs, we first performed live-cell imaging using a laser microirradiation assay in cells individually expressing GFP-tagged ZNF proteins. Rapid accumulation of these ZNF proteins at DNA damage stripes was observed for ZNF184, ZNF22, ZNF43, ZNF423, and ZNF428, indicating potential involvement in early DDR signaling. In contrast, ZNF738 failed to localize to the damaged regions, suggesting differential functional responses among these family members (Fig. 2A and Supplementary Fig. S2A–E). To further validate the recruitment of ZNF proteins to site-specific DSBs, we employed the LacI-FokI endonuclease system in U2OS cells, in which a repetitive DNA-associated clustered DSBs is induced at a defined genomic locus by doxycycline-inducible expression of an mCherry-FokI fusion protein tethered to a LacO array [50]. Notably, only GFP-ZNF184 robustly colocalized with mCherry-FokI foci, whereas the other tested ZNFs showed no accumulation (Fig. 2B and Supplementary Fig. S2F–J), indicating that ZNF184 exhibits a specific and reproducible recruitment to bona fide DSBs. We next confirmed ZNF184’s association with DNA damage sites using γH2AX, a well-established marker of DSBs. Immunofluorescence microscopy revealed clear colocalization of both ectopically expressed and endogenous ZNF184 with γH2AX foci following ionizing radiation (Fig. 2C and D), further supporting its role in the DSB response. To identify the domain responsible for ZNF184’s recruitment to DSBs, we generated a series of deletion mutants encompassing key structural features of the protein, including its zinc finger (ZnF) domains (Fig. 2E). These constructs were evaluated for their ability to localize to DNA lesions. Among the deletion mutants, only the construct lacking the ZnF domain (designated D4) failed to accumulate at DNA damage sites (Fig. 2F–H). Collectively, these results demonstrate that ZNF184 is uniquely recruited to sites of DSBs among the tested ZNF proteins, and that its ZnF domain is essential for this function.

### ZNF184 Expression induces accumulation of DSBs in ALL by suppressing homologous recombination

**Figure 3.**
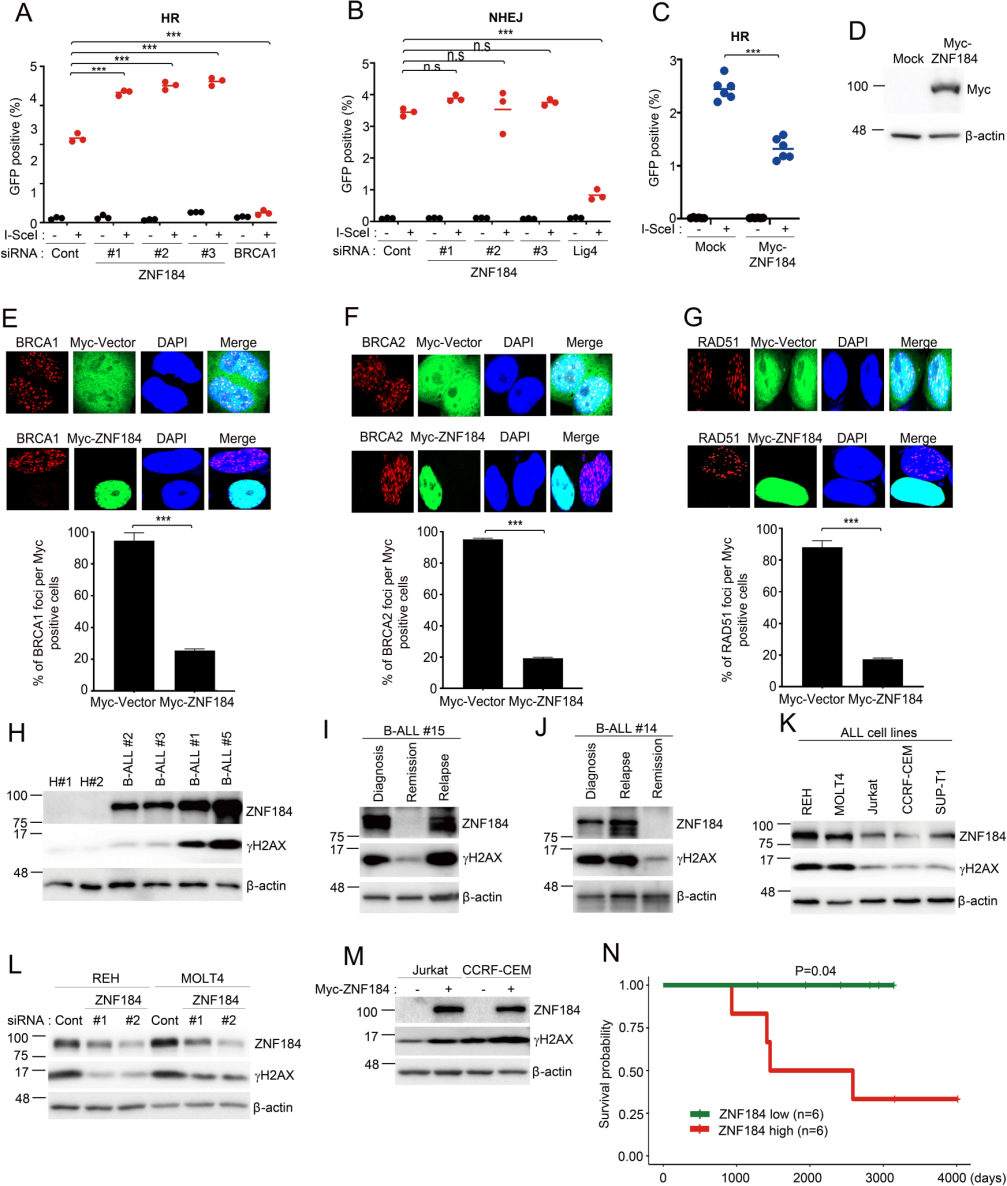
Expression of ZNF184 protein induces the accumulation of DSBs in ALL. (**A**) Measurement of HR capacity in DR-GFP reporter U2OS cells. U2OS cells harboring the DR-GFP reporter were treated with the indicated siRNAs. Two days later, GFP expression was accessed by flow cytometry. (**B**) Measurement of NHEJ capacity in EJ5-GFP reporter U2OS cells. U2OS cells harboring the EJ5-GFP reporter were treated with the indicated siRNAs. Two days later, GFP expression was accessed by flow cytometry. (**C, D**) Measurement of HR capacity in DR-GFP reporter U2OS cells. U2OS cells harboring the DR-GFP reporter were transfection with the indicated expression plasmids. Two days later, GFP expression was accessed by flow cytometry. Data represents the mean ± SD of two independent experiments. *P*-value was calculated based on two-way ANOVA in (A-C, ^***^P < .001, n.s., nonsignificant). (**E**–**G**) The mock and Myc-ZNF184 protein expression vectors were transfected into U2OS cells. After 48 h of transfection, the transfected U2OS cells were exposed to 5 Gy of ionizing radiation. At 4 h after the irradiation, the cells were fixed and stained with anti-Myc, BRCA1 (**E**), BRCA2 (**F**), and RAD51 (**G**) antibody. DAPI was used as a nuclear indicator. Data represent the mean ± SD of two independent experiments. *P*-value was calculated based on one-way ANOVA in (***P < .001). (**H**) Western blot analysis of ZNF184 and γH2AX levels using ALL patient tissue samples. H#1 and H#2 represent healthy donor. B-ALL, Precursor B-cell. (**I, J**) Western blot analysis of ZNF184 and γH2AX levels in patient derived ALL serial samples. (**K**) Western blot analysis of ZNF184 and γH2AX levels in untreated ALL cell lines. (**L**) REH and MOLT4 ALL cells were transfected with control siRNA or two different siZNF184s. At 48h after transfection, the transfected cell lysates were immunoblotted with the indicated antibodies. (**M**) Jurkat and CCRF-CEM ALL cells were transfected with control and ZNF184 expression plasmid. At 48 h after transfection, the transfected cell lysates were immunoblotted with the indicated antibodies. (**N**) Kaplan–Meier survival plot illustrating the association between *ZNF184* expression levels and overall survival in ETV6-RUNX1-positive primary samples from the TARGET-ALL-P2 dataset. Patients were stratified into high-expression (red line) and low-expression (green line) groups based on the mean *ZNF184* expression level (*n* = 6 per group). Statistical significance was determined using the log-rank test (*P* = .04).

To elucidate the functional significance of ZNF184 in DSB-induced DDR, we employed the DR-GFP and EJ5-GFP reporter assays to quantify HR and NHEJ efficiencies. ZNF184 knockdown (KD) enhanced HR activity, NHEJ efficiency was not significantly altered upon ZNF184 deficiency (Fig. 3A and B, and Supplementary Fig. S3A–D). Conversely, ZNF184 overexpression suppressed HR repair efficiency (Fig. 3C and D, and Supplementary Fig. S3E). Notably, this regulatory effect occurred independently of RAD51, BRCA1, or 53BP1 expression changes (Supplementary Fig. S3A and B), suggesting unchanged expression of HR factors excludes indirect HR suppression via transcriptional regulation. To confirm these findings and rule out off-target effects of siRNA-mediated ZNF184 KD, we performed rescue experiments using a siRNA-resistant ZNF184 WT expression vector. Restoring ZNF184 expressions in knockdowned cells reduced HR efficiency, further supporting its inhibitory role in HR repair (Supplementary Fig. S3F and G). Given these observations, we next sought to determine whether ZNF184 interferes with the recruitment of HR machinery to DSBs. We focused on BRCA1, BRCA2, and RAD51, critical mediators of HR. Following IR, cells overexpressing ZNF184 exhibited significantly reduced BRCA1, BRCA2, and RAD51 foci formation compared to control cells (Fig. 3E–G), whereas ZNF184 deficiency significantly increased BRCA1, BRCA2, and RAD51 accumulation at DSBs (Supplementary Fig. S3H–J), supporting the hypothesis that ZNF184 disrupts BRCA1, BRCA2, and RAD51 recruitment to DSBs, thereby impairing HR-mediated repair. Next, we analyzed ZNF184 expression in ALL patient samples. ZNF184 expression was significantly higher in ALL patient samples compared to healthy donors (Fig. 3H), suggesting its potential involvement in hematologic malignancies. Serial sample analysis from two patients showed dynamic changes in ZNF184 levels, with increased expression observed at diagnosis and relapse, but not during remission (Fig. 3I and J). We also examined ZNF184 expressions in ALL cell lines, including REH, MOLT4, Jurkat, CCRF-CEM, and SUP-T1, and observed differential expression levels across these cell lines (Fig. 3K). Notably, increased γH2AX levels were detected in cells with high ZNF184 expression (Fig. 3H–K), supporting its role as a negative regulator of the DDR. To validate these findings, we manipulated ZNF184 expression in ALL cell lines. *ZNF184* KD in REH and MOLT4 cell lines (high ZNF184 expression) reduced γH2AX levels (Fig. 3L). Conversely, ZNF184 overexpression in Jurkat and CCRF-CEM cell lines (low ZNF184 expression) significantly increased γH2AX levels (Fig. 3M). In REH and MOLT4 cells, which exhibit relatively high ZNF184 expression, we observed correspondingly high levels of TRIM28 and H3K9me3. These cells also showed elevated basal γH2AX levels, consistent with increased endogenous DNA damage signaling (Supplementary Fig. S3K). Kaplan–Meier analysis using the TARGET-ALL-P2 cohort revealed that high ZNF184 expression was associated with poorer overall survival in *ETV6::RUNX1*-positive patients (Fig. 3N), suggesting that ZNF184 is not only a mechanistic driver of genomic instability but also a potential prognostic biomarker in ALL.

### ZNF184 Modulates DNA damage repair and sensitizes ALL cells to PARPi

**Figure 4.**
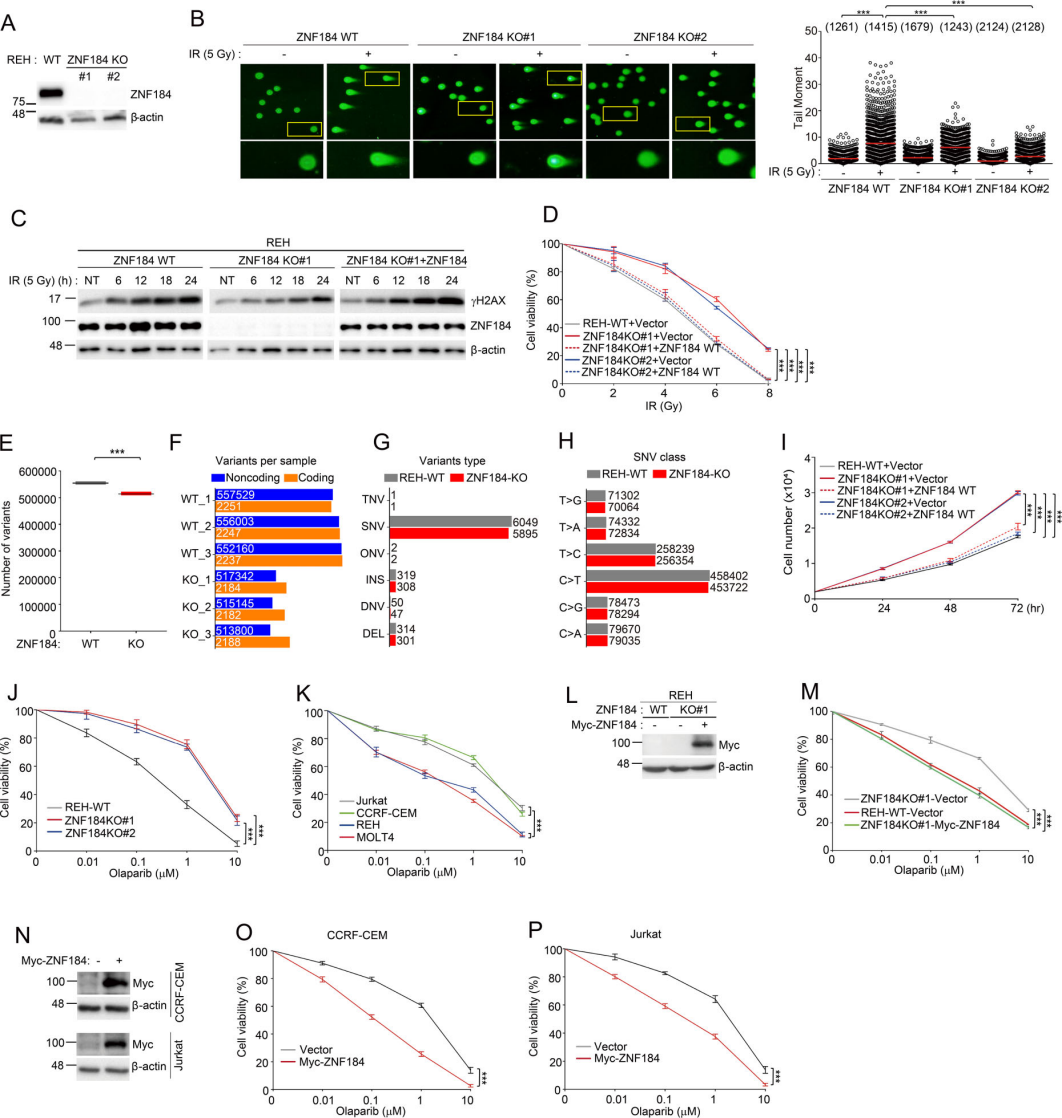
ZNF184 is a key factor for maintenance of genomic integrity and cell survival. (**A**) Western blot using *ZNF184* WT or KO REH cells. (**B**) Visualization of a neutral comet assay results showing IR-induced DNA DSBs in *ZNF184* WT or KO REH cells. At 2 h after exposure to 5 Gy of IR, the tail moment was measured as an endpoint to assess DNA DSBs. Each graph represents distribution of the tail moment. The numbers above each sample indicate the number of nuclei analyzed (*n*). One-way ANOVA was used to determine statistical significance (***P < .001). (**C**) *ZNF184* WT and KO REH cells were transduced with either pLenti CMV GFP-2A-Puro vector control or a vector expressing ZNF184 WT. After 2 days of transduction, the cells were exposed to 5 Gy of IR. And then, REH cells were collected at indicated times. Cell lysates were immunoblotted with the indicated antibodies. (**D**) *ZNF184* WT and KO REH cells were transduced with either pLenti CMV GFP-2A-Puro vector control or a vector expressing ZNF184 WT. After 2 days of transduction, 5000 cells were plated and treated with increasing dose of IR (0, 2, 4, 6, and 8 Gy). And then, cell viability was assessed after 3 days. (**E**) A boxplot comparing the number of relative variants between ZNF184 WT and ZNF184 KO (corrected using Welch’s *t*-test, ***P < .001). (**F**) A bar plot comparing the number of variants per sample between ZNF184 WT and ZNF184 KO. (**G**) A bar plot representing the relative number of coding region variants for each variant type between ZNF184 WT and ZNF184 KO. TNV, triple nucleotide variants; SNV, single nucleotide variants; ONV, oligo-nucleotide variants; INS, insertion; DNV, double nucleotide variants; DEL, deletion. (**H**) A bar plot depicting the relative count of SNV types between ZNF184 WT and ZNF184 KO. SNV, single nucleotide variant. (**I**) Cell proliferation assay comparing ZNF184 WT, KO, and ZNF184-rescued KO REH cells. A total of 5000 cells were seeded, and viable cells were counted at the indicated time points. (**J**) Viability of *ZNF184* WT and KO REH cells following treatment with an Olaparib. Five-thousand cells were plated and treated with increasing concentrations of Olaparib (0, 0.01, 0.1, 1, and 10 μM). The number of cells was counted culture with Olaparib for 4 days. (**K**) Viability of indicated cells following treatment with an Olaparib. Five-thousand cells were plated and treated with increasing concentrations of Olaparib (0, 0.01, 0.1, 1, and 10 μM). The number of cells was counted culture with Olaparib for 4 days. The mock and Myc-ZNF184 protein expression vectors were transfected into REH *ZNF184* WT, KO#1 (**L**), CCRF-CEM, and Jurkat (**N**) cells. Cell lysates were immunoblotted with the indicated antibodies. (**M, O**, and **P**) Viability of indicated cells following treatment with a Olaparib. Five-thousand cells were plated and treated with increasing concentrations of Olaparib (0, 0.01, 0.1, 1, and 10 μM). The number of cells was counted culture with Olaparib for 4 days. Results are presented as the average of three independent experiments. Statistical analysis was performed by two-way ANOVA and data represents the mean ± S.D (D, I, J, K, M, O, P, ***P < .001).

To delineate the functional role of ZNF184 in the DDR, particularly in the context of IR-induced DSBs, we established *ZNF184* KO cells using CRISPR/Cas9-mediated gene editing in REH ALL cells with *ZNF184*-specific guide RNAs (Supplementary Fig. S4A). Successful KO was confirmed through western blotting and DNA sequencing (Fig. 4A and Supplementary Fig. S4B–G). To investigate whether ZNF184 influences DNA repair efficiency, we performed alkaline comet assays in *ZNF184* WT and KO cells following 5 Gy of IR. The comet tail moment, measured 2 h post-irradiation, was significantly reduced in *ZNF184* KO cells, suggesting enhanced DNA repair capacity in the absence of *ZNF184* (Fig. 4B). This observation was further supported by quantification of γH2AX western data, a surrogate marker for unrepaired DSBs. *ZNF184* KD via RNA interference also led to diminished γH2AX levels (Supplementary Fig. S5A). Consistently, *ZNF184* KO cells exhibited a marked reduction in γH2AX signal compared to WT cells following IR (Supplementary Fig. S5B), indicating more efficient resolution of DSBs, reinforcing the notion that ZNF184 negatively regulates HR repair. To verify the causative role of ZNF184, we reintroduced ZNF184 WT into KO cells via lentiviral transduction, which significantly increased IR-induced γH2AX levels (Fig. 4C), indicating suppressed DNA repair activity. To elucidate the biological relevance of ZNF184-modulated DDR and DSB repair, we analyzed the radiosensitivity of *ZNF184* WT, KO, or ZNF184 WT rescue KO REH ALL cell lines. *ZNF184* depletion reduced IR sensitivity compared to WT REH cells, and restoration of ZNF184 expression by lentiviral transduction significantly increased the sensitivity (Fig. 4D and Supplementary Fig. S5C and D), indicating that downregulation of ZNF184 enhanced DDR or DSB repair more efficiently. The WGS analysis revealed that ZNF184 KO cells harbored fewer genomic variants than WT counterparts (Fig. 4E and F), indicating improved genome maintenance. Single nucleotide variants (SNVs) predominated, with C > T transitions representing the most frequent variant class (Fig. 4G and H). These findings suggest a potential connection between ZNF184 and the maintenance of genomic stability. Consistent with enhanced DNA repair, *ZNF184*-deficient cells exhibited accelerated proliferation compared to WT cells, and restoration of ZNF184 expression by lentiviral transduction significantly reduced the cell proliferation (Fig. 4I and Supplementary Fig. S5E–G), suggesting that loss of ZNF184 promotes cell survival and growth under genotoxic conditions. Given that ZNF184 expression appears to suppress HR-mediated repair, we evaluated its impact on sensitivity to Olaparib, which are synthetically lethal in HR-deficient contexts. As shown in Fig. 4J, *ZNF184* depletion reduced Olaparib sensitivity compared to WT REH cells. Baseline expression levels of ZNF184 were quantified across various ALL cell lines; REH and MOLT4 displayed high ZNF184 expression, whereas Jurkat and CCRF-CEM exhibited low expression (Fig. 3K). Notably, cell lines with elevated ZNF184 levels were significantly more sensitive to the Olaparib than those with low expression (Fig. 4K). Furthermore, re-expression of ZNF184 in KO REH cells restored their sensitivity to Olaparib (Fig. 4L and M). Similarly, the ectopic expression of ZNF184 in Jurkat and CCRF-CEM cells enhanced their response to Olaparib (Fig. 4N–P). Collectively, these findings identify ZNF184 as a negative regulator of HR and a determinant of sensitivity to PARP inhibition in ALL. ZNF184 overexpression compromises DNA repair fidelity, thereby creating a therapeutically exploitable vulnerability to Olaparib.

### ZNF184 Interacts with tripartite motif containing 28 (TRIM28) and promotes its recruitment to sites of DNA damage

**Figure 5.**
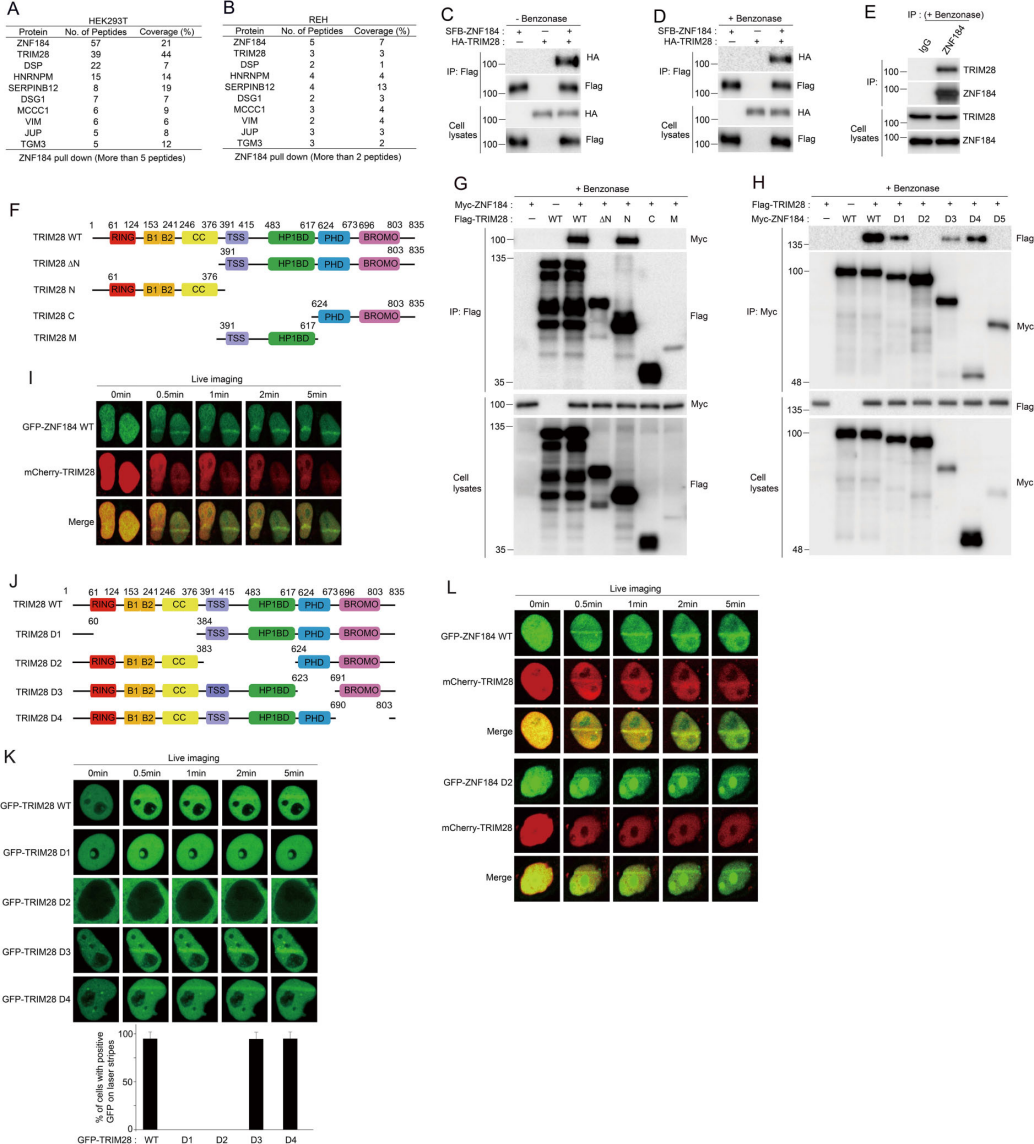
ZNF184 interacts with TRIM28 and promotes its recruitment to sites of DNA damage. (**A, B**) The list of proteins identified by mass spectrometric analyses of ZNF184 protein. The top nine proteins copurified with ZNF184 are shown. (**C, D**) The interaction between exogenous ZNF184 and TRIM28. The indicated plasmids were transfected into REH cells. After 24 h, the transfected cell lysates were immunoprecipitated (IP) using an anti‐Flag antibody with or without benzonase and subjected to western blotting analysis using the indicated antibodies. The bottom panel shows equal volumes of cell lysates immunoblotted with the indicated antibodies. (**E**) The interaction between endogenous ZNF184 and TRIM28. Immunoprecipitation reactions were performed using rabbit IgG or anti‐ZNF184 antibodies with benzonase and subjected to western blotting analysis using the indicated antibodies. The bottom panel shows equal volumes of cell lysates immunoblotted with the indicated antibodies. (**F**) Diagram of WT TRIM28 and serial deletion mutants. The numbers indicate the amino acid residues. RING, interesting new gene; B1, B-box 1; B2, B-box 2; CC, coiled-coil; TSS, TRIM-specific sequence; HP1BD, heterochromatin protein 1-binding domain; PHD, plant homeodomain; BROMO, bromodomain. (**G**) Myc‐ZNF184 and either Flag‐TRIM28 WT or its serial deletion mutants were co‐transfected into REH cells. The cell lysates were immunoprecipitated with the anti‐Flag antibody and then immunoblotted with the indicated antibodies. (**H**) Flag‐TRIM28 and either Myc‐ZNF184 WT or its serial deletion mutants were co‐transfected into REH cells. The cell lysates were immunoprecipitated with the anti‐Myc antibody and then immunoblotted with the indicated antibodies. (**I**) The recruitment kinetics of GFP-ZNF184 and mCherry-TRIM28 translocation to DNA damage sites. (**J**) Diagram of WT TRIM28 and internal deletion mutants. Numbers indicate amino acids. (**K**) HeLa cells were transfected with GFP-TRIM28 WT or deletion mutant expression plasmids, and 24 h later, the cells were treated with laser microirradiation (top panel). The cells with positive GFP expression on the laser stripes are presented in the bar graph (bottom panel). The results represent the average of two independent experiments. The error bars indicate the SD for the cells transfected with each expression plasmid. (**L**) Cotransfection of mCherry-TRIM28 WT with each of GFP-ZNF184 WT, or GFP-ZNF184 D2 mutant plasmid in HeLa cells. After 24 h, the transfected cells were microirradiated and the recruitment of these proteins to laser strips was examined by live cell imaging.

To explore the molecular mechanisms of ZNF184 protein, we performed tandem affinity purification using HEK 293T and REH ALL cell lines overexpressing SFB‐ZNF184. Mass spectrometry analysis identified several ZNF184‐binding proteins (Fig. 5A and B). Among the candidates, TRIM28 was selected for further investigation as the most promising interactor, showing 44% and 3% sequence coverage in the respective purifications (Fig. 5A and B). To validate the interaction between ZNF184 and TRIM28, immunoprecipitation assays were performed using cells overexpressing SFB‐ZNF184 and HA‐TRIM28 with or without benzonase treatment. The results showed that ZNF184 interacts with TRIM28 independently of benzonase (Fig. 5C and D). Additionally, endogenous immunoprecipitation with benzonase confirmed the ZNF184–TRIM28 interaction (Fig. 5E), indicating that this binding is independent on DNA or RNA. To identify the critical domains of TRIM28 responsible for binding to ZNF184, a series of TRIM28 deletion mutants were generated (Fig. 5F), and each mutant was cotransfected with SFB-ZNF184 WT into REH cells, followed by coimmunoprecipitation. The analysis revealed that the RBCC domain of TRIM28 is responsible for interacting with ZNF184 (Fig. 5G). Additionally, the KRAB domain of ZNF184 was found to bind TRIM28 (Figs 5H and 2E). It has been reported that TRIM28 localizes DNA damage sites [51–53]. In line with this, our study demonstrates that ZNF184 also accumulates at DNA damage sites. To explore the interplay between these two proteins, we investigated whether ZNF184 influences TRIM28’s recruitment and translocation to DNA damage regions. To examine the kinetics of ZNF184 and TRIM28 recruitment to DNA damage sites, we performed real-time imaging of protein accumulation at microirradiation-induced laser stripes. Fig. 5I shows that GFP-ZNF184 and mCherry-TRIM28 proteins were recruited to DNA damage sites within 0.5 min post-microirradiation. To identify the region of TRIM28 responsible for this translocation, we transfected TRIM28 WT and a series of internal deletion mutants (Fig. 5J). As shown in Fig. 5K, both TRIM28 WT and the D3 and D4 deletion mutants are localized to DNA damage sites, whereas the D1 mutant, which lacks the ZNF184-binding region, and the D2 mutant, which remained in the cytosol, did not. These results suggest that the ZNF184-binding region is crucial for TRIM28 accumulation at DNA damage sites. Furthermore, we observed that ZNF184 WT colocalized with TRIM28 WT at laser stripes in HeLa cells, whereas the ZNF184 D2 mutant, which lacks the TRIM28-binding region, failed to do so (Fig. 5L and Supplementary Fig. S6A). These findings imply that ZNF184 promotes TRIM28 recruitment to DNA damage sites through direct interaction with TRIM28.

### ZNF184 Modulates TRIM28 phosphorylation and chromatin remodeling to regulate DSB repair

**Figure 6.**
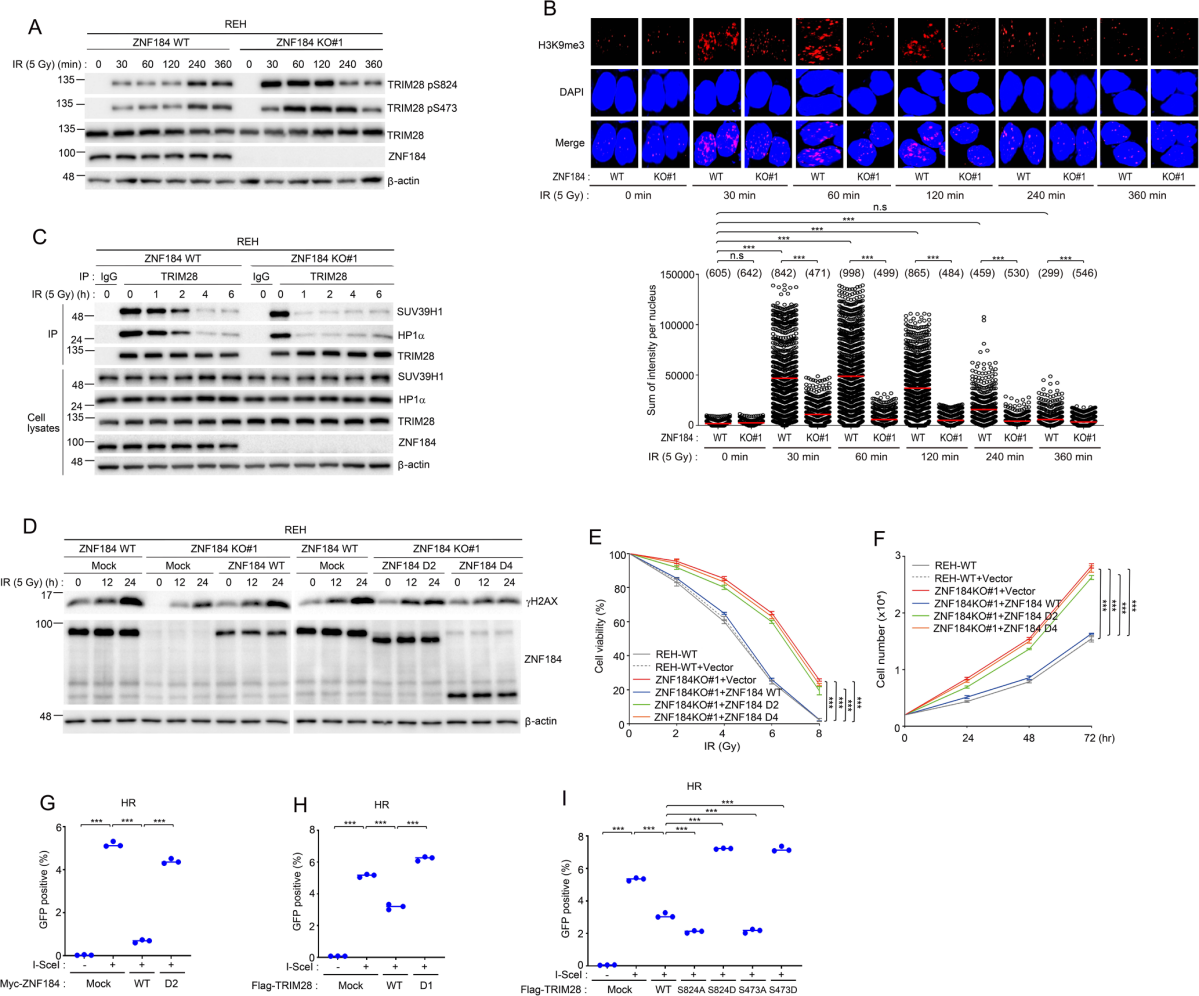
ZNF184 modulates TRIM28 phosphorylation and chromatin decondensation to facilitate DSB repair. (**A**) *ZNF184* WT and KO REH cells were exposed to 5 Gy of IR. And then, REH cells were collected at indicated times. Cell lysates were immunoblotted with the indicated antibodies. (**B**) *ZNF184* WT and KO REH cells were exposed to 5 Gy of IR. And then, the cells were fixed and stained with anti-H3K9me3 antibody at indicated times. H3K9me3 intensity was determined by immunofluorescence. DAPI was used as a nuclear indicator. The numbers above each sample indicate the number of nuclei analyzed (*n*). (**C**) *ZNF184* WT and KO REH cells were exposed to 5 Gy of IR. And then, REH cells were collected at indicated times. Immunoprecipitation reactions were performed using rabbit IgG or anti‐ZNF184 antibodies and subjected to western blotting analysis using the indicated antibodies. The bottom panel shows equal volumes of cell lysates immunoblotted with the indicated antibodies. (**D**) *ZNF184* WT and KO REH cells were transduced with either pLenti CMV GFP-2A-Puro vector control, a vector expressing ZNF184 WT, D2, or D4. After 2 days of transduction, the cells were exposed to 5 Gy of IR. And then, REH cells were collected at indicated times. Cell lysates were immunoblotted with the indicated antibodies. *ZNF184* WT and KO REH cells were transduced with control, ZNF184 WT, D2, or D4 were subjected to graded IR doses (0–8 Gy) to assess viability (**E**), and proliferation was measured by seeding 5000 cells and counting viable cells at indicated time points (**F**). Measurement of HR capacity in DR-GFP reporter U2OS cells. U2OS cells harboring the DR-GFP reporter were transfected with the indicated plasmid, Myc-ZNF184 WT or D2 mutant (**G**), Flag-TRIM28 WT or D1 mutant (**H**), Flag-TRIM28 WT or S824A, S824D, S473A, S473D (**I**). After 2 days of transfection, GFP expression was accessed by flow cytometry. Data represents the mean ± SD of two independent experiments. *P*-value was calculated based on one-way (B, G, H, I) or two-way (E and F) ANOVA in (***P < .001, n.s., nonsignificant).

Upon induction of DSBs, the DDR pathway is activated to preserve genomic integrity. TRIM28 is rapidly phosphorylated at serine 473 (pS473) and serine 824 (pS824) in response to genotoxic stress, primarily mediated by ATM kinase [51, 52]. These phosphorylation events play a critical role in modulating TRIM28’s interaction with heterochromatin, promoting its dissociation from chromatin and thereby facilitating the recruitment of DNA repair machinery to the damaged sites [53–55]. To investigate whether ZNF184 plays a role in modulating TRIM28 phosphorylation, we examined the levels of TRIM28 phosphorylation at serine 473 (pS473) and serine 824 (pS824) in *ZNF184* WT and KO cells following IR. At early post-irradiation time points, *ZNF184*-deficient cells exhibited significantly elevated phosphorylation at both S473 and S824 relative to WT controls, indicating an accelerated or hyperactive initial DDR signal (Fig. 6A). Strikingly, at later time points, pS473 and pS824 levels in *ZNF184* KO cells declined below those observed in WT cells. These findings suggest that ZNF184 regulates the temporal dynamics of TRIM28 phosphorylation, potentially controlling chromatin relaxation kinetics and efficient DNA repair. We further evaluated the chromatin state by assessing H3K9me3, a repressive histone mark associated with heterochromatin. At 30 min post-IR, *ZNF184* KO cells exhibited a notable reduction in H3K9me3 levels compared to WT cells (Fig. 6B), indicating defective heterochromatin re-establishment. Since TRIM28 cooperates with HP1 and SUV39H1 to establish H3K9me3 at DSB-flanking regions, we next investigated whether the TRIM28–HP1–SUV39H1 complex assembly is perturbed in *ZNF184*-deficient cells. Coimmunoprecipitation assays revealed diminished interactions between TRIM28 and both HP1 and SUV39H1 in *ZNF184* KO cells following IR, compared to WT cells (Fig. 6C), suggesting that ZNF184 is required for proper recruitment or stabilization of the TRIM28 repressive complex at sites of damage. To further delineate the functional domains of ZNF184 necessary for genome maintenance, we generated lentiviral constructs expressing full-length ZNF184 (ZNF184-WT) and two deletion mutants (ZNF184-D2: deleted TRIM28 binding domain and ZNF184-D4: deleted the DNA damage localization domain) and introduced them into *ZNF184* KO#1 or *ZNF184* KO#2 REH cells. Restoration of DNA repair signaling was evaluated by monitoring γH2AX signals following IR. Cells expressing ZNF184-WT, but not the deletion mutants, exhibited enhanced γH2AX signals (Fig. 6D and Supplementary Fig. S7A). Moreover, only ZNF184-WT expression conferred increased sensitivity to IR-induced stress (Fig. 6E and Supplementary Fig. S7B). Intriguingly, ZNF184-WT expression rescues the hyper-proliferative phenotype of KO cells (Fig. 6F and Supplementary Fig. S7C). In HR reporter assays, full-length ZNF184 significantly reduced HR efficiency, consistent with its role as a negative regulator of HR. In contrast, the D2 mutant did not reduce HR activity, indicating that this domain alone is insufficient to mediate HR suppression (Fig. 6G and Supplementary Fig. S7D and E).

To directly establish a functional connection between ZNF184-TRIM28-mediated chromatin regulation and HR, we performed HR reporter assays in cells expressing endogenous TRIM28 together with ectopically expressed TRIM28 variants. Expression of WT TRIM28 significantly suppressed HR activity, whereas a TRIM28 D1 mutant lacking the ZNF184-binding region failed to repress HR, indicating that the interaction between ZNF184 and TRIM28 is essential for HR suppression (Fig. 6H and Supplementary Fig. S7F and G). We next examined whether TRIM28 phosphorylation contributes to HR regulation. Cells expressing nonphosphorylatable TRIM28 mutants (S473A or S824A) exhibited reduced HR efficiency, whereas phospho-mimetic mutants (S473D or S824D) showed increased HR activity (Fig. 6I and Supplementary Fig. S7H and I). These results demonstrate that ZNF184-dependent modulation of TRIM28 phosphorylation and chromatin state is directly linked to HR repair outcomes.

Collectively, these data establish ZNF184 as a critical regulator of TRIM28 phosphorylation and chromatin remodeling at DSBs. By promoting proper assembly of the TRIM28/HP1/SUV39H1 complex and sustaining DDR signaling, ZNF184 suppresses repair and thereby contributes to enhanced genomic instability in hematologic malignancies.

### ZNF184 Modulates DDR in primary ALL cells and confers therapeutic vulnerability

**Figure 7.**
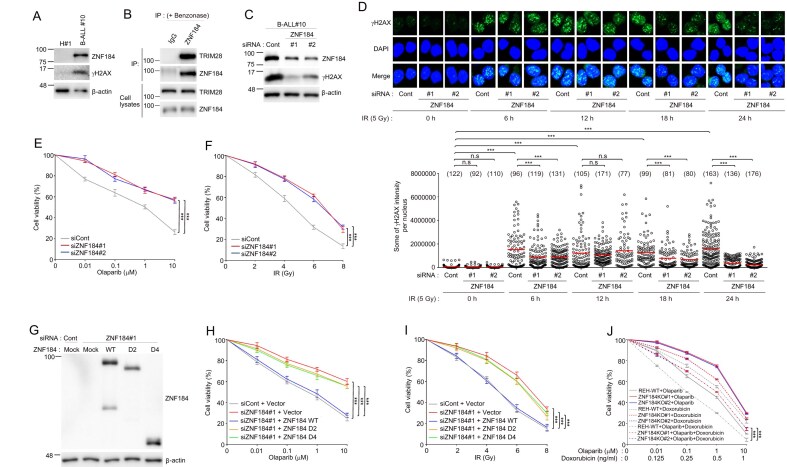
ZNF184 modulates DDR in primary ALL cells. (**A**) Western blot analysis of ZNF184 and γH2AX levels using ALL patient tissue sample. H#1 represents healthy donor. B-ALL, precursor B-cell. (**B**) The interaction between endogenous ZNF184 and TRIM28 in primary B-ALL cells. Immunoprecipitation reactions were performed using rabbit IgG or anti‐ZNF184 antibodies with benzonase and subjected to western blotting analysis using the indicated antibodies. The bottom panel shows equal volumes of cell lysates immunoblotted with the indicated antibodies. (**C**) Primary B-ALL cells were transfected with control siRNA or two different siZNF184. Forty-eight hours after transfection, the transfected cell lysates were immunoblotted with the indicated antibodies. (**D**) Control or *ZNF184* siRNA transfected primary B-ALL cells were exposed to 5 Gy of IR. And then, the cells were fixed and stained with anti-γH2AX antibody at indicated times. γH2AX intensity was determined by immunofluorescence. DAPI was used as a nuclear indicator. The numbers above each sample indicate the number of nuclei analyzed (*n*). (**E**) Viability of control or *ZNF184* siRNA transfected primary B-ALL cells following treatment with an Olaparib. After 2 days of transfection, 5000 cells were plated and treated with increasing concentrations of Olaparib (0, 0.01, 0.1, 1, and 10 μM). The number of cells was counted culture with Olaparib for four days. (**F**) Viability of control or *ZNF184* siRNA transfected primary B-ALL cells following treatment with IR. After 2 days of transfection, 5000 cells were plated and treated with increasing dose of IR (0, 2, 4, 6, and 8 Gy). And then, cell viability was assessed after 3 days. (**G**) Control or *ZNF184* siRNA transfected primary B-ALL cells were transduced with either pLenti CMV GFP-2A-Puro vector control, a vector expressing ZNF184 WT, D2 or D4. After 2 days of transduction, the transduced cell lysates were immunoblotted with the indicated antibodies. (**H**) Control or *ZNF184* siRNA transfected primary B-ALL cells were transduced with either pLenti CMV GFP-2A-Puro vector control, a vector expressing ZNF184 WT, D2, or D4. After 2 days of transduction, 5000 cells were plated and treated with increasing concentrations of Olaparib (0, 0.01, 0.1, 1, and 10 μM). The number of cells was counted culture with Olaparib for 4 days. (**I**) Control or *ZNF184* siRNA transfected primary B-ALL cells were transduced with either pLenti CMV GFP-2A-Puro vector control, a vector expressing ZNF184 WT, D2 or D4. Two days of post-transduction, 5000 cells were plated and treated with increasing dose of IR (0, 2, 4, 6, and 8 Gy). And then, cell viability was assessed after 3 days. (**J**) Viability of *ZNF184* WT and KO REH cells following treatment with indicated materials. After 2 days of transfection, 5000 cells were plated and treated with increasing concentrations of Olaparib (0, 0.01, 0.1, 1, and 10 μM) with or without doxorubicin (0, 0.125, 0.25, 0.5, and 1 ng/ml). And then, cell viability was assessed after 3 days. Data represents the mean ± SD of two independent experiments. *P*-value was calculated based on one-way (D) or two-way (E, F, and H–J) ANOVA in (***P < 001, n.s., nonsignificant).

To explore the clinical relevance of ZNF184 in B-ALL, we investigated its expression and functional role in primary patient-derived cells. Peripheral blood mononuclear cells (PBMCs) were isolated from a newly diagnosed B-ALL patient and compared to PBMCs from healthy donors. Western blot analysis revealed that ZNF184 protein expression was markedly elevated in the patient sample relative to healthy control (Fig. 7A), consistent with our prior observations in B-ALL primary cells. Previously, we demonstrated the interaction between ZNF184 and TRIM28 in the REH ALL cell line. We further validated this finding in primary patient-derived cells, confirming that ZNF184 also interacts with TRIM28 in patient samples through immunoprecipitation (Fig. 7B). To functionally investigate the role of ZNF184 in DNA repair, we employed loss-of-function and rescue strategies in the primary B-ALL PBMCs. siRNA-mediated ZNF184 KD resulted in a substantial decrease of γH2AX signals and increase in cell viability following treatment with either IR or the Olaparib (Fig. 7C–F). Conversely, ectopic expression of ZNF184 WT in ZNF184-depleted PBMCs restored sensitivity to both Olaparib and IR-induced genotoxic stress (Fig. 7G–I).

### ZNF184 Expression confers sensitivity to combined PARP inhibition and genotoxic chemotherapy in ALL cells

Given that ZNF184 suppresses HR-mediated DNA repair, we hypothesized that inhibition of PARP in ALL cells expressing ZNF184 would synergize with genotoxic chemotherapeutic agents. Consistent with this hypothesis, REH cell lines expressing WT ZNF184 (ZNF184 WT), which are highly sensitive to the PARP inhibitor Olaparib, also exhibited increased sensitivity to doxorubicin, a frontline chemotherapeutic agent commonly used in ALL treatments. Cotreatment with Olaparib and doxorubicin resulted in a pronounced proliferative defect in ZNF184 WT REH cells (Fig. 7J), indicating a synergistic cytotoxic effect. These findings support a mechanistic model in which elevated ZNF184 expression compromises HR efficiency, thereby enhancing reliance on PARP-dependent DNA repair pathways. Consequently, PARP inhibition induces synthetic lethality in ZNF184-expressing cells, a vulnerability that is further amplified by the addition of DNA-damaging agents such as doxorubicin.

## Discussion

ALL is a malignancy marked by the clonal expansion of immature lymphoid precursors. This process is frequently accompanied by genomic and epigenomic perturbations that compromise DDR fidelity. Although the functional role of DDR dysregulation in ALL pathogenesis has not been fully delineated, accumulating evidence implicates DDR defects in leukemogenesis, relapse, and therapeutic resistance [56–58].

Our study provides mechanistic and translational insights into this process by identifying ZNF184, a previously uncharacterized ZNF, as a critical modulator of HR repair, chromatin remodeling, and therapeutic response in ALL.

We found that ZNF184 expression is significantly elevated in ALL patient samples, with dynamic changes observed across diagnosis, remission, and relapse. High ZNF184 expression levels strongly correlated with disease burden and poor clinical outcomes in *ETV6::RUNX1*-positive ALL. These observations position ZNF184 as a potential dynamic biomarker for disease monitoring and prognosis prediction. Notably, its expression was consistently higher in leukemic blasts compared to healthy BM, highlighting its functional relevance in leukemia biology.

Mechanistic investigations revealed that ZNF184 is rapidly recruited to DSBs through its zinc finger domain. It colocalizes with γH2AX and plays a specific role in regulating HR repair. ZNF184 acts as a negative regulator of HR, impairing BRCA1 recruitment to damaged chromatin and thereby reducing repair efficiency. This function was not mediated through transcriptional suppression of canonical DDR genes such as RAD51 or 53BP1, suggesting a direct mechanistic interface at damage sites. Interestingly, ZNF184 depletion enhanced HR repair efficiency and reduced DSB marker accumulation after IR, while KO cells exhibited decreased mutation rates and genomic instability, indicating that ZNF184 serves a dual role as both a gatekeeper and a suppressor of excessive recombination.

A key discovery of this study is the interaction between ZNF184 and the chromatin remodeling factor TRIM28, a protein implicated in heterochromatin regulation and transcriptional silencing. ZNF184 binds TRIM28 via its KRAB domain, facilitating TRIM28 recruitment to DNA lesions and regulating its phosphorylation at serine residues S473 and S824-modifications required for heterochromatin relaxation and proper chromatin reassembly. Loss of ZNF184 impaired the reformation of the TRIM28–HP1–SUV39H1 complex, leading to defective chromatin compaction following DNA repair. These findings underscore ZNF184’s role as a scaffold and modulator of chromatin dynamics during the DDR.

From a therapeutic standpoint, our data show that ZNF184 defines a state of “BRCAness” in ALL cells, conferring heightened sensitivity to PARPi. ALL cells with high ZNF184 expression were markedly sensitive to Olaparib, an effect reversible upon ZNF184 KD and reproducible via ectopic overexpression. Furthermore, combining Olaparib with doxorubicin, a DNA-damaging agent, resulted in synergistic cytotoxicity in ZNF184-expressing cells, including patient-derived blasts. These results suggest that ZNF184 may serve as a predictive biomarker for PARPi responsiveness and a potential therapeutic target in combination with conventional chemotherapy.

Notably, ZNF184’s role in DDR intersects with clinically relevant resistance mechanisms. Among the drugs commonly used in pediatric ALL therapy, 6-mercaptopurine acts as a pro-drug that forms thioguanine nucleotides after intracellular metabolism within lymphoid cells. These thioguanine nucleotides integrate into the DNA, leading to thioguanine–thymine mismatches and subsequent cell death [59]. However, defects in the mismatch repair system can disrupt this therapeutic mechanism, contributing to drug resistance [60]. Mutations in mismatch repair genes are frequently associated with ALL relapses, likely due to resistance to 6-mercaptopurine therapy [61]. Error-prone DNA damage repair is also reported to enable the leukemic cells to escape CD22-directed therapy with Inotuzumab ozogamicin. Inotuzumab ozogamicin is an antibody-drug conjugate that delivers calicheamicin to cells expressing CD22, with calicheamicin binding to the DNA minor groove to induce DSBs [62]. These observations highlight the urgent need for DDR modulators to restore repair fidelity and sensitize resistant leukemic clones-roles that ZNF184 may fulfill.

In summary, ZNF184 is a previously unrecognized but functionally critical regulator of HR suppression, chromatin remodeling, and therapeutic sensitivity in ALL. Its dual roles-as a mechanistic effector of DDR and a clinically relevant biomarker-underscore its potential for translation into targeted therapeutic strategies. Future studies should investigate the broader applicability of ZNF184-guided PARPi therapy across hematologic malignancies and explore TRIM28-mediated chromatin remodeling as a therapeutic axis for intervention.

## Supplementary Material

gkag486_Supplemental_Files

## Data Availability

The previously published bulk RNA-seq (GEO: GSE116229) and single-cell RNA-seq (GEO: GSE130116) data utilized in this study are publicly available. The cell line WGS data generated during the current study have been deposited in the NCBI Sequence Read Archive (SRA, https://www.ncbi.nlm.nih.gov/sra) under BioProject accession PRJNA1332923. Other raw sequencing data from clinical samples generated and analyzed during the current study are available from the corresponding author on reasonable request. Mass spec proteomics data has been deposited in PRIDE (https://www.ebi.ac.uk/pride/archive) under accession number PXD066624. All other data supporting the findings of this study are also available from the corresponding author upon reasonable request.
