# Supplementary material for: ZNF184 negatively regulates HR repair and predicts poor prognosis in acute lymphoblastic leukemia
Source: Nucleic Acids Res. 2026 May 20;54(10):gkag486. doi: 10.1093/nar/gkag486 (PMC13187848; doi:10.1093/nar/gkag486)
Supplement: gkag486_Supplemental_Files [file gkag486_supplemental_files.zip › 120526010701_Supplementary_Information.docx]

**Supplementary Information for**

# ZNF184 Negatively Regulates HR Repair and Predicts Poor Prognosis in Acute Lymphoblastic Leukemia

Won Chan Hwang^1,13^, Hee Young Ju^2,13^, Kibeom Park^1,13^, Eun Jung Kwon^3,13^, Eun Seop Seo^2,4,5,1^^3^, Yuheon Chung^6^, Byung-Gyu Kim^6^, Kyungjae Myung^6,7^, Dong Min Lim^8^, Yun Hak Kim^9,10,11*^, Keon Hee Yoo^2,4,12,*^, and Hongtae Kim^1,*^

^*^Corresponding authors. Email: khtcat@unist.ac.kr, hema2170@skku.edu, yunhak10510@pusan.ac.kr

**This PDF file includes:**

Supplementary Figures and legends (Supplementary Figure S1 to S7)

**Supplementary Figures**

Supplementary Figure S1


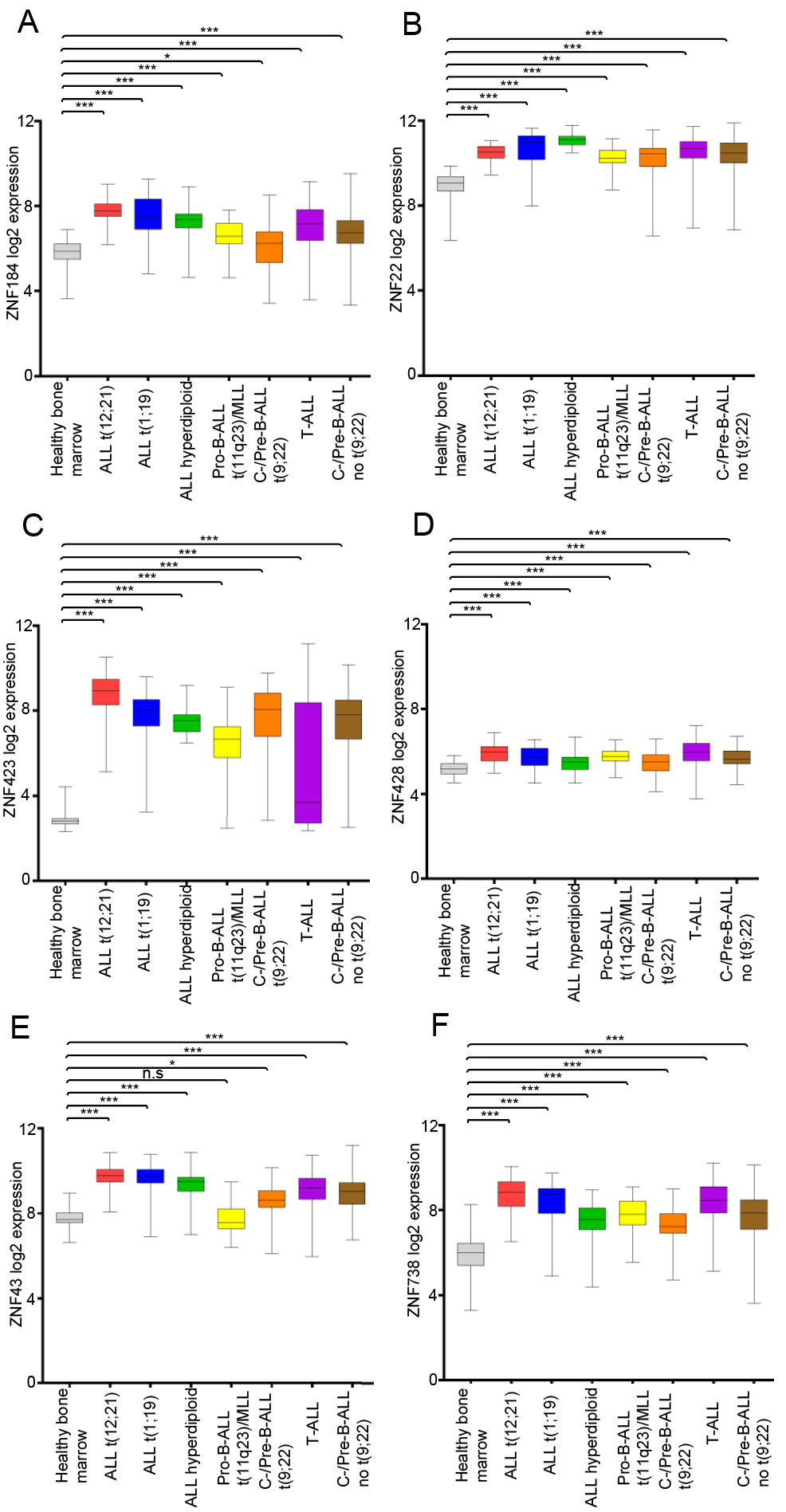


**Supplementary Figure S1. Comparative analysis of selected ZNF gene expression between Healthy controls and ALL subtypes.**

**(A-F)** *ZNF* genes expression in cohort by RNA-seq data using the Cancer Genome Atlas (TCGA) data ([www:bloodspot.eu/](https://xena.ucsc.edu/)). The *P*-value was calculated by Wilcoxon rank-sum test (**P* < 0.01, ****P* < 0.001, n.s. non-significant).

Supplementary Figure S2

**
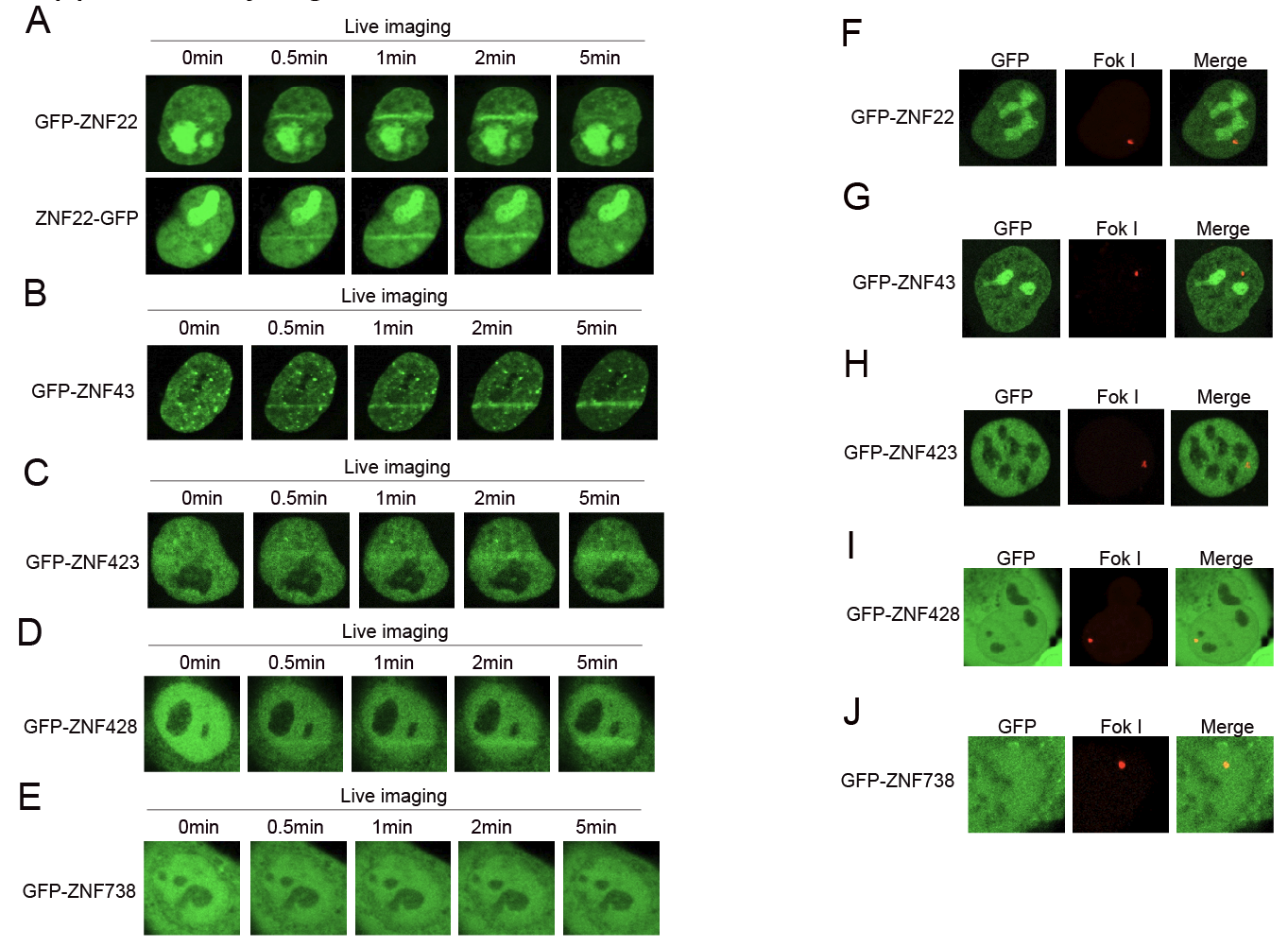
**

**Supplementary Figure S2. ZNF proteins increased in ALL are not involved in double strand breaks.**

**(A-E)** Hela cells expressing GFP-ZNF22, ZNF22-GFP (**A**), GFP-ZNF43 (**B**), GFP-ZNF423 (**C**), GFP-ZNF428 (**D**), and GFP-ZNF738 (**E**) were subjected to laser microirradiation. The laser stripes were examined at the indicated time points. **(F-J)** mCherry-LacI-FokI was co-transfected with indicated GFP-tagged expression vectors into U2OS-DSB reporter cells. After 48 h, live cell imaging was performed with confocal microscopy.

Supplementary Figure S3

**
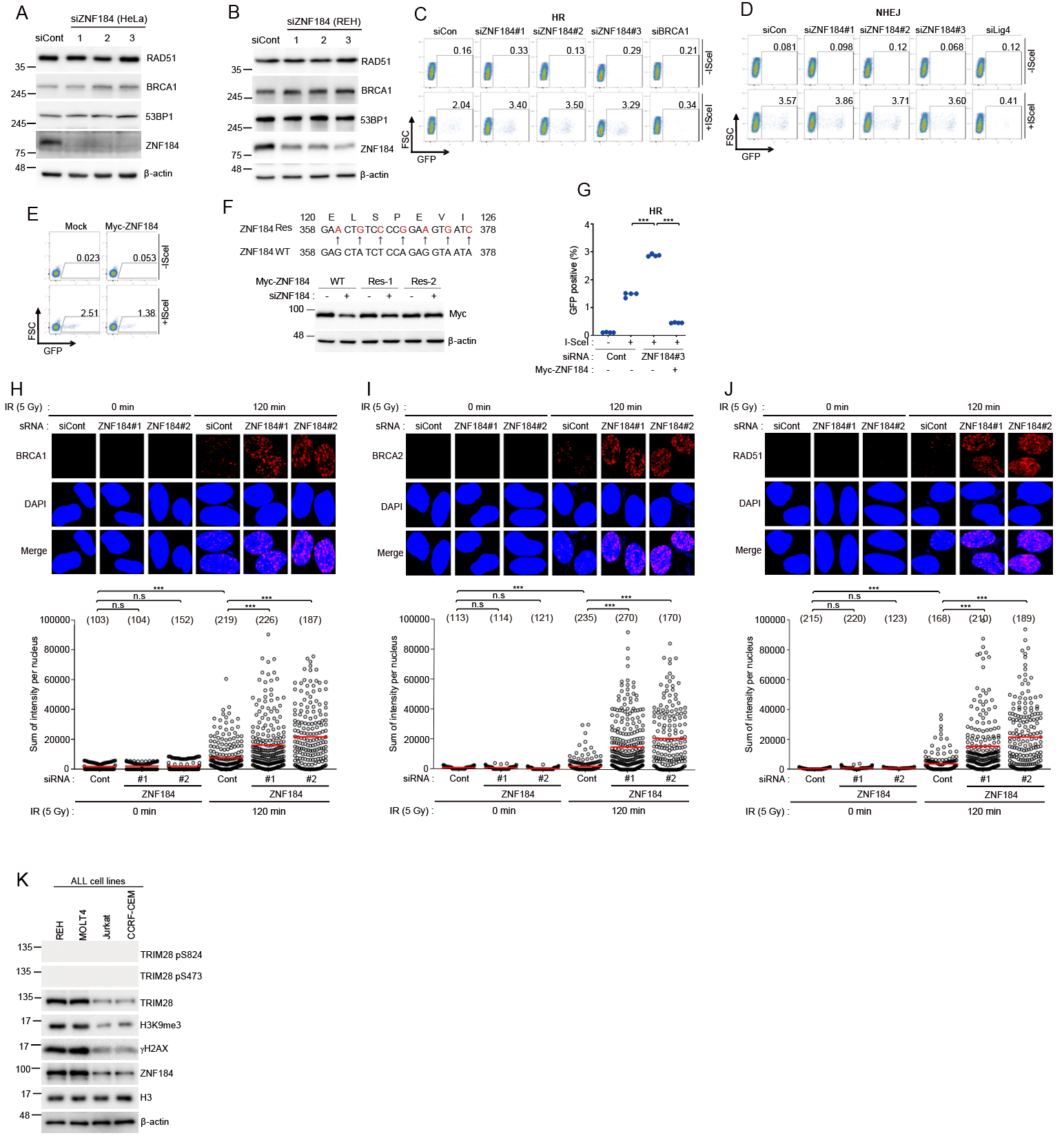
**

**Supplementary Figure S3. Deficiency of ZNF184 activates the HR repair pathway, but NHEJ is not significant.**

**(A and B)** Measurement of HR or NHEJ pathway involved proteins in Hela or REH cells. Hela or REH cells were treated with the indicated siRNAs. Cell lysates of indicated siRNAs were immunoblotted with indicated antibodies. **(C and D)** Measurement of HR capacity in DR-GFP (**C**) or EJ5-GFP (**D**) reporter U2OS cells. **(E)** The mock and Myc-ZNF184 protein expression vectors were transfected into DR-GFP U2OS cells. Two days later, GFP expression was accessed by flow cytometry. **(F)** The sequences of the siRNA-resistant ZNF184 WT cDNA showing silent mutation (ZNF184-RES). For protein expression of ZNF184-RES, HEK293T cells were transfected siRNA for 24 h and were transfected ZNF184 plasmid as indicated for 24 h. And then whole cell lysates were prepared for western blotting to confirm that the ZNF184-RES is resistant to the ZNF184 siRNA treatment. **(G)** Measurement of homologous recombination capacity in DR-GFP reporter U2OS cells. U2OS cells harboring the DR-GFP reporter were treated with the indicated siRNAs, followed by transfection with the indicated expression plasmids. Two days later, GFP expression was accessed by flow cytometry. **(H – J)** REH cells were transfected with control or *ZNF184* siRNAs. After 24 h, 1 X 10^6^ cells were plated, transfected cells were exposed to 5 Gy IR. After 2 h of the irradiation, the cells were fixed and stained with anti-BRCA1 **(H)**, BRCA2 **(I)**, and RAD51 **(J)** antibody. DAPI was used as a nuclear indicator. **(K)** Western blot analysis of indicated antibodies in untreated ALL cell lines. All results are presented as three independent experiments. Error bars indicate standard deviation. *P*-value was calculated based on one-way ANOVA in **(G - J)** (^***^*P* < 0.001, n.s. non-significant).

Supplementary Figure S4

**
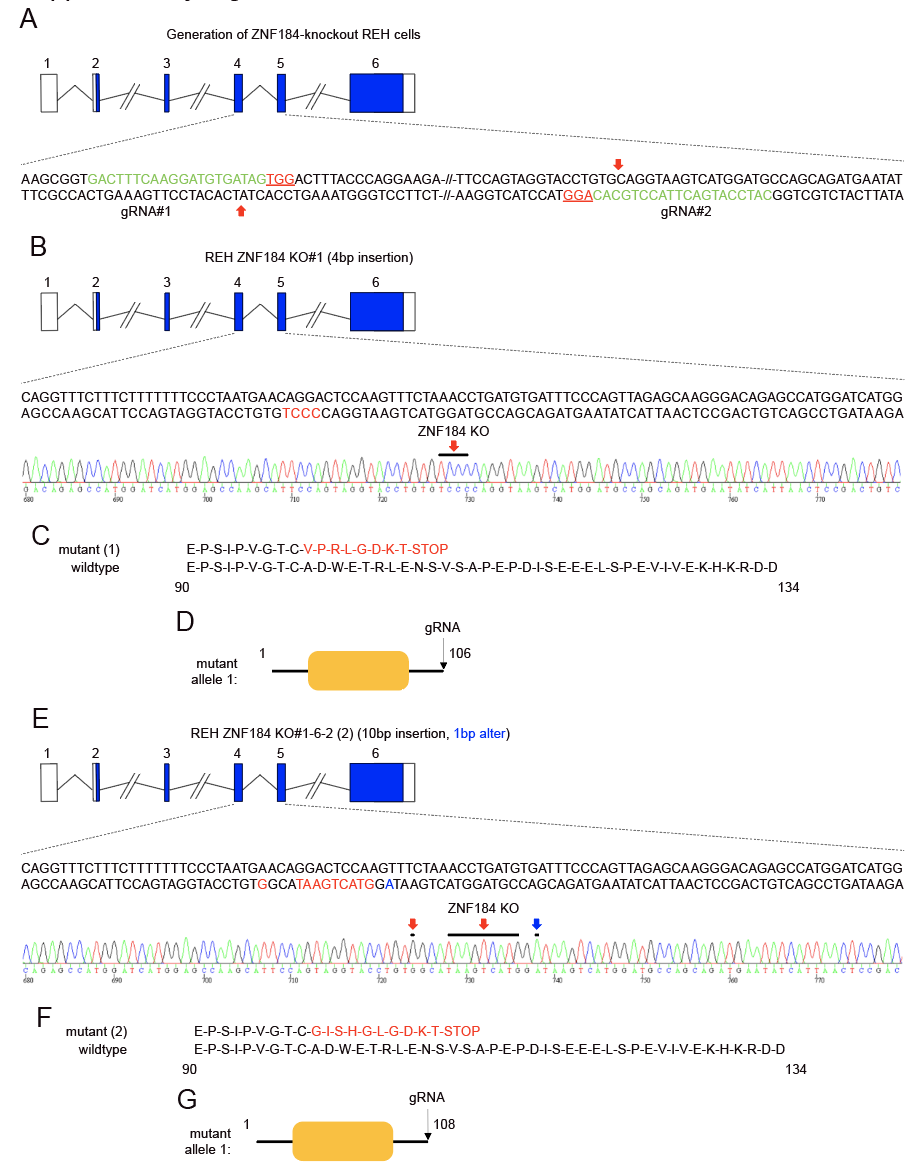
**

**Supplementary Figure S4. Generation of *ZNF1184* KO REH cell lines.**

**(A)** Human *ZNF184* genomic structure and guide RNAs in exon 4 and 5. Red arrow: cut site, green: target sequence for guide RNA, Red (underlined): PAM sequence. **(B)** The sequence of the first mutant allele in ZNF184 KO REH cells (clone 1) is aligned with the sequence of ZNF184 from control cells. **(C)** The predicted protein sequence in the region of ZNF184 affected by the mutation in the first mutant allele in ZNF184 KO REH cells in **(B)**. The alteration in the amino acid sequence is highlighted in red. **(D)** The predicted protein size in the region of ZNF184 is affected by the mutation in the first mutant allele in ZNF184 KO REH cells in **(B)**. Yellow box is KRAB domain. **(E-G)** Same as **(B-D)** except that they refer to the second mutated allele in compound homozygous ZNF184 KO REH cells (clone 2).

Supplementary Figure S5


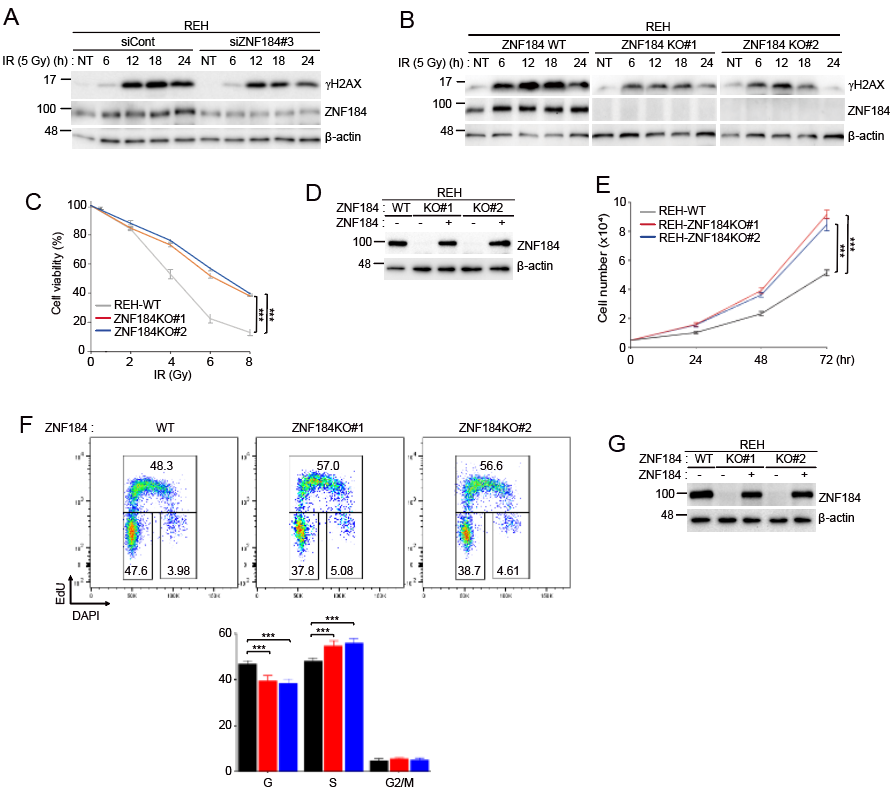


**Supplementary Figure S5. Deficiency of ZNF184 improved DNA repair by IR-induced DNA damage.**

**(A)** REH cells were transfected with control or *ZNF184* siRNAs#3. After 24 h of transfection, 1 X 10^6^ cells were plated, transfected cells were exposed to 5 Gy of IR, and were collected at indicated time points. Cell lysates of indicated siRNAs were immunoblotted with indicated antibodies. **(B)** ZNF184 WT and KO REH cells were exposed to 5 Gy of IR. And then, REH cells were collected at indicated times. Cell lysates were immunoblotted with the indicated antibodies. **(C)** Survival of *ZNF184* WT and KO REH cells were exposed to dose dependent of IR. 5000 cells were plated and treated with increasing dose of IR (0, 2, 4, 6, and 8 Gy). The number of cells was counted culture with IR for 3 days. **(D)** ZNF184 WT and KO REH cells were transduced to pLenti CMV GFP 2A Puro-Vector or -ZNF184 WT virus. After 2 days of transduction, cell lysates were immunoblotted with the indicated antibodies. **(E)** Cell proliferation of *ZNF184* WT and KO REH cells. 5000 cells were plated, and the number of viable cells was counted at indicated time points. **(F)** Cell cycle analysis of *ZNF184* WT and KO REH cells using flow cytometry. Cell cycles were evaluated with EdU and DAPI staining. **(G)** ZNF184 WT and KO cell lines were recovery by overexpressing empty vector or ZNF184 via lentivirus. After 2 days of transduction, cell lysates were immunoblotted with the indicated antibodies. Data represents the mean ± SD of two independent experiments. *P*-value was calculated based on two-way ANOVA in (**C, E, F,** ^***^*P* < 0.001).

Supplementary Figure S6

**
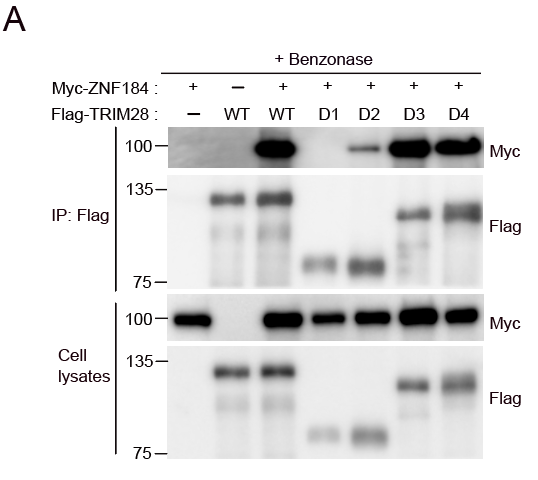
**

**Supplementary Figure S6. ZNF184 bound to the RBCC domain of TRIM28.**

**(A)** Myc‐ZNF184 and either Flag‐TRIM28 WT or its serial deletion mutants were co‐transfected into REH cells. The cell lysates were immunoprecipitated with the anti‐Flag antibody and then immunoblotted with the indicated antibodies.

Supplementary Figure S7


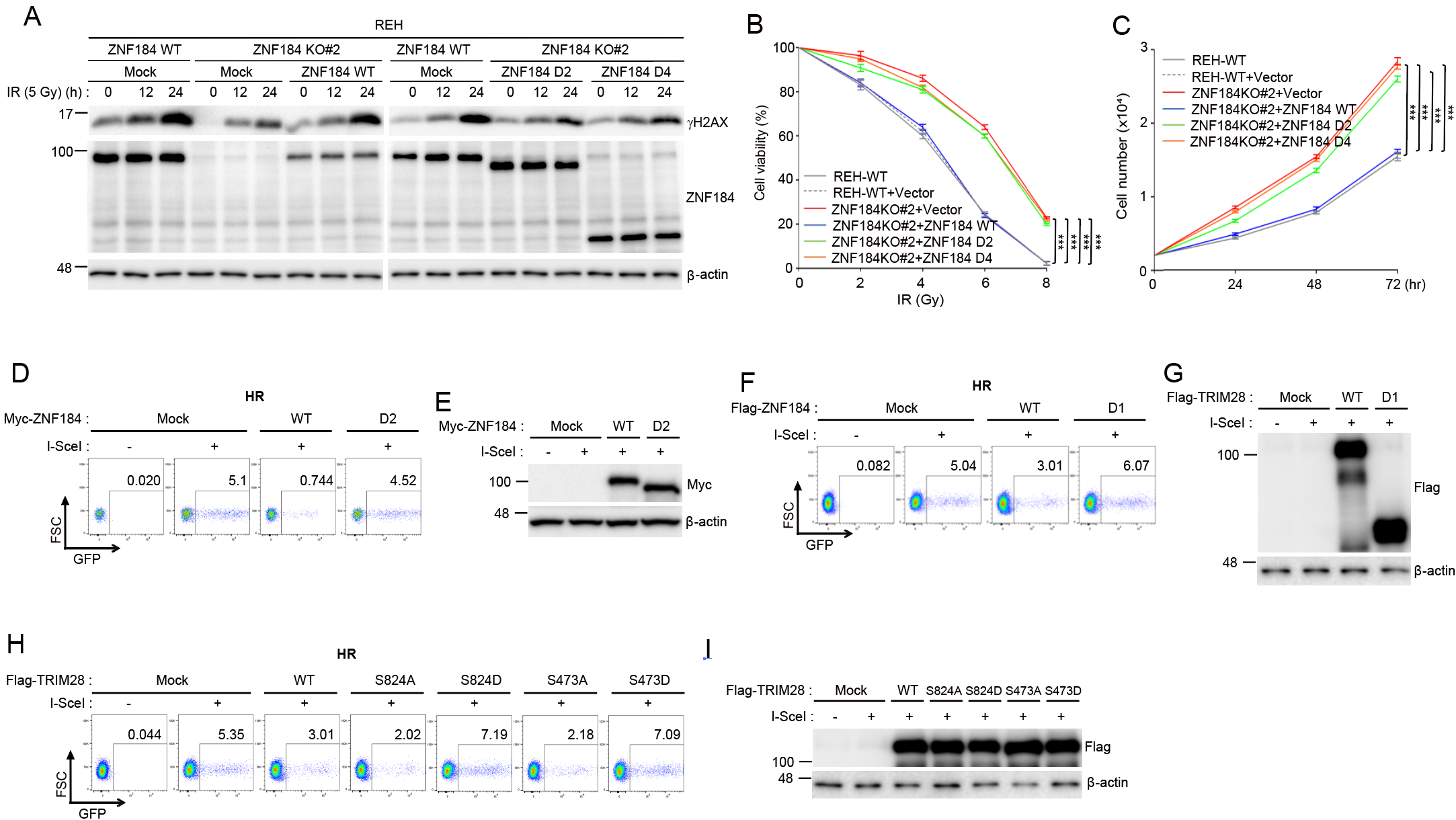


**Supplementary Figure S7. ZNF184-TRIM28 axis directly modulates HR repair in reporter assay.**

**(A)** *ZNF184* WT and KO REH cells were transduced with either pLenti CMV GFP-2A-Puro vector control, a vector expressing ZNF184 WT, D2 or D4. After 2 days of transduction, the cells were exposed to 5 Gy of IR. And then, REH cells were collected at indicated times. Cell lysates were immunoblotted with the indicated antibodies. **(B and C)** *ZNF184* WT and KO REH cells were transduced with control, ZNF184 WT, D2, or D4 were subjected to graded IR doses (0–8 Gy) to assess viability **(B)**, and proliferation was measured by seeding 5,000 cells and counting viable cells at indicated time points **(C)**. **(D-I)** Measurement of homologous recombination capacity in DR-GFP reporter U2OS cells. U2OS cells harboring the DR-GFP reporter were transfected with the indicated plasmid, Myc-ZNF184 WT or D2 mutant **(D and E)**, Flag-TRIM28 WT or D1 mutant **(F and G)**, Flag-TRIM28 WT or S824A, S824D, S473A, S473D **(H and I)**. Two days later, GFP expression was accessed by flow cytometry and western blot. Data represents the mean ± SD of two independent experiments. *P*-value was calculated based on two-way **(B, C)** ANOVA in (****P* < 0.001).
