# Supplementary material for: ZNF184 negatively regulates HR repair and predicts poor prognosis in acute lymphoblastic leukemia
Source: Nucleic Acids Res. 2026 May 20;54(10):gkag486. doi: 10.1093/nar/gkag486 (PMC13187848; doi:10.1093/nar/gkag486)
Supplement: gkag486_Supplemental_Files [file gkag486_supplemental_files.zip › Supplementary Information_Tables legernd.docx]

**Supplementary Information for**

# ZNF184 Negatively Regulates HR Repair and Predicts Poor Prognosis in Acute Lymphoblastic Leukemia

Won Chan Hwang^1,13^, Hee Young Ju^2,13^, Kibeom Park^1,13^, Eun Jung Kwon^3,13^, Eun Seop Seo^2,4,5,1^^3^, Yuheon Chung^6^, Byung-Gyu Kim^6^, Kyungjae Myung^6,7^, Dong Min Lim^8^, Yun Hak Kim^9,10,11*^, Keon Hee Yoo^2,4,12,*^, and Hongtae Kim^1,*^

^*^Corresponding authors. Email: khtcat@unist.ac.kr, hema2170@skku.edu, yunhak10510@pusan.ac.kr

**Supplementary Table 1. List of differentially expressed ZNF genes from bulk RNA sequencing analysis.**

This table summarizes 54 differentially expressed ZNF genes (DEGs) identified among 562 ZNF family members, including 30 upregulated and 24 downregulated genes in patient samples relative to healthy controls.

**Supplementary Table 2. Upregulated ZNF genes identified in ETV6::RUNX1 fusion-positive ALL by single-cell RNA-seq.**

Analysis of single-cell RNA sequencing data identified 26 upregulated ZNF differentially expressed genes (DEGs) among 505 genes specifically in ETV6::RUNX1 fusion-positive acute lymphoblastic leukemia cases.

**Supplementary Table 3. Overlapping upregulated ZNF genes identified from bulk and single-cell RNA sequencing analyses.**

Seven ZNF genes (ZNF184, ZNF22, ZNF423, ZNF428, ZNF43, ZNF726, and ZNF738) were commonly upregulated across both datasets.

**Supplementary Table 4. List of siRNA, primer, and gRNA sequences used in this study.**

The sequences of all siRNAs, primers, and gRNAs used in this study are provided.
